# Interleukin-1β Drives Disease Progression in Arrhythmogenic Cardiomyopathy

**DOI:** 10.1016/j.jacbts.2026.101542

**Published:** 2026-05-05

**Authors:** Vinay R. Penna, Junedh M. Amrute, Morgan Engel, Emily A. Shiel, Waleed Farra, Elisa N. Cannon, Colleen Leu-Turner, Pan Ma, Ana Villanueva, Haewon Shin, Alekhya Parvathaneni, Joanna Jager, Carlos Bueno-Beti, Angeliki Asimaki, Kory J. Lavine, Jeffrey E. Saffitz, Stephen P. Chelko

**Affiliations:** a Center for Cardiovascular Research, Division of Cardiology, Department of Medicine, Washington University in St Louis School of Medicine, St Louis, Missouri, USA; b Department of Biomedical Sciences, Florida State University College of Medicine, Tallahassee, Florida, USA; c Creighton University, School of Medicine, Emergency Medicine, Phoenix, Arizona, USA; d Department of Pathology and Immunology, Washington University in St Louis School of Medicine, St Louis, Missouri, USA; e Cardiovascular and Genomics Research Institute, School of Health and Medical Sciences, City St George’s, University of London, London, United Kingdom; f Department of Developmental Biology, Washington University in St Louis School of Medicine, St Louis, Missouri, USA; g Department of Pathology, Beth Israel Deaconess Medical Center and Harvard Medical School, Boston, Massachusetts, USA

**Keywords:** arrhythmogenic cardiomyopathy, cardioimmunology, CCR2^+^ macrophages, myocardial inflammation

## Abstract

Arrhythmogenic cardiomyopathy (ACM) is a genetic form of heart failure that affects 1 in 5,000 people globally and is caused by mutations in cardiac desmosomal genes including *PKP2, DSP*, and *DSG2.* Individuals with ACM suffer from ventricular arrhythmias, sudden cardiac death, and heart failure. There are few effective treatments and heart transplantation remains the best option for many affected individuals. Here we performed single nucleus RNA sequencing and spatial transcriptomics on myocardial samples from patients with ACM and control donors. We identified disease-associated spatial niches characterized by coexistence of fibrotic and inflammatory cell types and failing cardiac myocytes. The inflammatory-fibrotic niche colocalized to areas of cardiac myocyte loss and comprised *FAP* (fibroblast activation protein) and *POSTN* (periostin) expressing fibroblasts, macrophages that expressed *NLRP3*, and nuclear factor κB activated genes. Using homozygous *Dsg2* mutant (*Dsg2*^mut/mut^) mice, we identified analogous populations of *Postn*-expressing fibroblasts and inflammatory macrophage populations that co-localized within diseased areas. Detailed single nucleus RNA-sequencing analysis of inflammatory macrophage subsets that were increased in ACM samples revealed high levels of *Il1b* expression. To delineate the possible benefit of targeting IL1B in ACM, we treated *Dsg2*^mut/mut^ mice with an anti-IL1B neutralizing antibody and observed attenuated fibrosis, reduced levels of inflammatory cytokines and chemokines, preserved cardiac function, and diminished conduction slowing and automaticity, key mechanisms of arrhythmogenesis. These results suggest that currently approved therapeutics that target IL1B or IL1 signaling may improve outcomes for patients with ACM.

## Highlights

• snRNAseq of human ACM hearts reveals increased proportions of distinct inflammatory myeloid cells and activated fibroblasts.
• Spatial transcriptomics analysis of human ACM reveals distinct spatial niches made up of inflammatory myeloid cells and activated fibroblasts in areas of tissue damage and fibrosis referred to as lesions.
• Inhibition of IL1B signaling using a neutralizing antibody leads to significant attenuation of ACM pathogenesis in the *Dsg2*^mut/mut^ mouse model of ACM.

## Visual abstract

**Figure:**
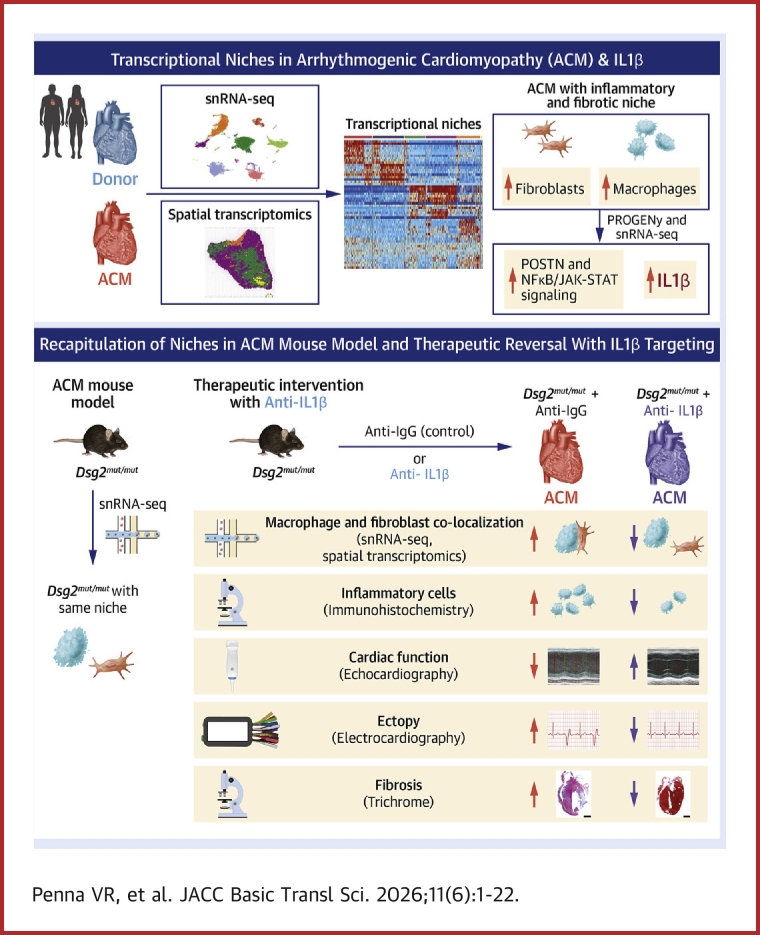

Arrhythmogenic cardiomyopathy (ACM) is a familial nonischemic heart disease, affecting 1:2,000 to 1:5,000 people, globally.^1^ It is among the leading causes of sudden cardiac death in young individuals due to malignant ventricular arrhythmias.^1^, ^2^, ^3^, ^4^ Individuals with ACM may also progress to end-stage heart failure. Currently, treatments such as antiarrhythmics, standard medical therapies for heart failure, and implantable cardioverter-defibrillators only temporize symptoms, and heart transplantation represents the only curative therapy.^5^^,^^6^

ACM is primarily caused by mutations in cardiac desmosomal genes.^7^ Whereas other cardiac cytoskeletal and ion transport genes may present with an ACM-like phenotype, most ACM cases stem from mutations in the desmosomal genes *PKP2, DSP,* and *DSG2*.^7^ How these mutations result in ACM is not clearly understood, but it is thought to involve abnormal nuclear factor κB (NFκB), Wnt/β-catenin, and Hippo signaling pathways.^8^, ^9^, ^10^ Recent studies have recognized that ACM is associated with a striking cardiac and systemic inflammatory response.^11^^,^^12^

Prominent myocardial fibrosis and inflammation have been observed in over 70% of autopsy samples from patients with ACM. Furthermore, patients with ACM display elevated serum levels of inflammatory cytokines.^1^^,^^2^^,^^11^^,^^13^ We have previously reported that signaling mediated via NFκB, a master regulator of the innate immune response, is activated in a mouse model of ACM harboring homozygous knock-in of a variant in the gene encoding the desmosomal protein, *Dsg2* (*Dsg2*^mut/mut^ mice).^14^ We observed that using a genetic approach to block NFκB signaling in cardiac myocytes alone is sufficient to prevent myocardial loss and fibrosis, preserve contractile function, and suppress arrhythmias in *Dsg2*^mut/mut^ mice.^15^ We also found that NFκB signaling in cardiac myocytes leads to a 5-fold increase in monocytes and macrophages expressing CCR2, a potent chemotactic molecule that has been implicated in adverse cardiac remodeling and fibrosis.^16^, ^17^, ^18^ Suppression of CCR2^+^ monocyte and macrophage recruitment to the heart was sufficient to halt progression of ACM pathology in *Dsg2*^mut/mut^ mice.^15^

Despite these recent insights, significant gaps remain in our understanding of how inflammatory monocyte and macrophage populations contribute to heart failure, myocardial inflammation and fibrosis, and arrhythmogenesis. Little is known regarding the cellular composition of ACM lesions or the key mediators of cardiac inflammation. Improved understanding of the cellular and transcriptomic landscape of ACM lesions and the aberrant cell signaling pathways used to drive tissue pathology will be critical to identify new therapeutic targets for this devastating disease.

To address these gaps in knowledge, we performed single nucleus RNA sequencing (snRNAseq) on myocardial samples from patients with clinically active ACM (n = 6; 3 patients with *DSP* variants and 3 patients with *PKP2* variants) and donor control subjects (n = 12; no history of heart disease). In addition, we performed spatial transcriptomic sequencing on ACM patient samples (n = 3; 2 patients with *PKP2* variants and 1 patient with a *DSP* variant) and donor control subjects (n = 2). Using these data, we deconvoluted the cellular landscape of ACM, identified ACM-associated disease signatures, and uncovered spatially restricted niches containing profibrotic fibroblasts and inflammatory macrophages that localized around areas of myocardial disease. Using an established mouse model of ACM (ie, *Dsg2*^mut/mut^ mice), we observed analogous cell populations and niches with enriched expression of inflammatory mediators, including IL1B. Our prior work demonstrated that myocardial IL1B levels were up-regulated (by 13-fold) in *Dsg2*^mut/mut^ mice compared to age-matched wild-type (WT) control mice.^19^ In this paper to establish a causative relationship between IL1 signaling and cardiac pathology, we treated *Dsg2*^mut/mut^ mice with a neutralizing antibody against IL1B and observed significant improvements in myocardial pathology and function. These findings highlight a role for targeting IL1 signaling in ACM.

## Methods

Additional detailed methods can be found in the Supplemental Appendix.

### Ethical approval for human samples

This study is compliant with all relevant ethical regulations and approved by Washington University School of Medicine Institutional Review Board (IRB no. 201104172). Each patient provided informed consent prior to tissue collection, and no compensation was provided for study participation. All patients have been deidentified.

### Ethical approval for animal studies

All experiments conformed to the Guide for the Care and Use of Laboratory Animals from the National Institute of Health (NIH publication no. 85–23, revised 1996). Animal study protocols were approved by the Florida State University (protocol code: 202000052; date of approval: February 10, 2021) and Washington University in St Louis (protocol code: D1600245, date of approval: December 2, 2020) Animal Care and Use Committee. Mice were housed in temperature-controlled rooms (20-22 °C) and humidity (40%-60%) with a 12-hour light/dark cycle and provided ad libitum access to standard rodent chow and water. Age-matched C57BL/6 mice served as WT control mice. The generation of *Dsg2*^mut/mut^ mice has been previously described.^1^

### Study design

The objective of this study was to elucidate the cellular and transcriptional environment of ACM and subsequently to better understand the role of inflammatory mediators (specifically IL1B) in ACM disease onset and progression. To assess the former, we performed snRNAseq and spatial transcriptomics using the 10x Genomics 5’v2 and Visium platforms, respectively. All human myocardial samples were previously frozen and obtained from the Tissue Cardiovascular Biobank and Repository at Washington University. For snRNAseq, we used all donor and ACM samples that met quality control cutoffs following library construction. For spatial transcriptomics, we selected tissue samples that met RNA quality control standards based on DV200 measurements (DV200 >40%) (Supplemental Table 1) prior to sectioning and sequencing.^20^^,^^21^ Whereas a DV200 ≥30% is recommended by the manufacturer (10x Genomics) for tissues broadly, more nuanced discussions with the onsite application field engineers led us to select a higher threshold of 40% for cardiac tissues given its relatively higher stiffness. For animal studies, we used the previously established *Dsg2*^mut/mut^ mouse model of ACM.^14^ To assess the presence of analogous myeloid and fibroblast populations in mice, we performed snRNAseq on sorted myeloid cells and fibroblasts from WT and *Dsg2*^mut/mut^ mice. The effects of IL1B blockade via anti-IL1B antibody on ACM disease progression in *Dsg2*^mut/mut^ mice was assessed via echocardiography, electrocardiography, histology, cytokine proteome arrays, and snRNAseq. Investigators were blinded to both genotype and drug treatment cohorts throughout experiments and analyses. Sample size (n = 5-9) was predetermined based on our prior publications,^14^^,^^15^^,^^19^ which demonstrated ≥4 mice was more than sufficient to show substantial cardiovascular and pathologic abnormalities between cohorts (ie, WT vs *Dsg2*^mut/mut^). Utilizing ≥4 mice/genotype/cohort/parameter, we were able to achieve at least 80% power to find a *P* value <0.05. No data were excluded from any analyses.

### Statistical analysis

Data are presented as mean ± SEM with sample size (n) provided within each figure legend for all in vivo studies. Comparison among more than 2 groups was determined by 1-way analysis of variance using Brown-Forsythe (skewed data) or Welch (unequal variances) analysis of variance. For comparisons between 2 groups, Welch’s *t*-test was used. Pearson correlation coefficient was used to determine the association between 2 continuous variables. For comparisons between 2 groups with unequal variances Welch's t-test was performed. To test normalcy of data such that parametric methods can be used to present and compare data, we used the Shapiro-Wilks test. We did not consider adjustment for multiple pairwise comparisons, which is a limitation to our statistical analysis. All results were repeated at least twice under the same or similar experimental conditions. All statistical analyses were done using GraphPad Prism (version 10, GraphPad Software, Dotmatics), and a 2-sided *P* < 0.05 was considered statistically significant.

### Neutralization of IL1B via anti-IL1B antibody in vivo

Mouse anti-IL1B and isotype control (mouse anti-immunoglobulin G [anti-IgG]) were generously provided by Novartis. WT and *Dsg2*^mut/mut^ mice were randomly assigned to either the isotype control (10 mg/kg/wk) or anti-IL1B treatment groups at 8 weeks of age (early intervention) or 16 weeks of age (late intervention). Mice in both studies were treated for 8 weeks with either 1 mg/kg/wk of mouse anti-IL1B or 10 mg/kg/wk of isotype control via hindlimb intramuscular injection. Mouse anti-IgG antibody was selected as placebo control given IgG antibodies have demonstrated to have the longest half-life of all immunoglobulin isotype antibodies and IgG is the predominant isotype.^22^ Furthermore, prior research has demonstrated anti-IgG isotype control antibodies can directly elicit an immune response; particularly at higher doses of anti-IgG (≥1 mg/kg). Thus, a 10-fold increase in isotype control (ie, 10 mg/kg/wk) was selected to ensure mouse anti-IgG antibody would accurately discriminate between results observed from anti-IL1B treated mice (1 mg/kg/wk).^22^, ^23^, ^24^, ^25^

## Results

### snRNAseq reveals the cellular landscape of ACM

**Figure 1:**
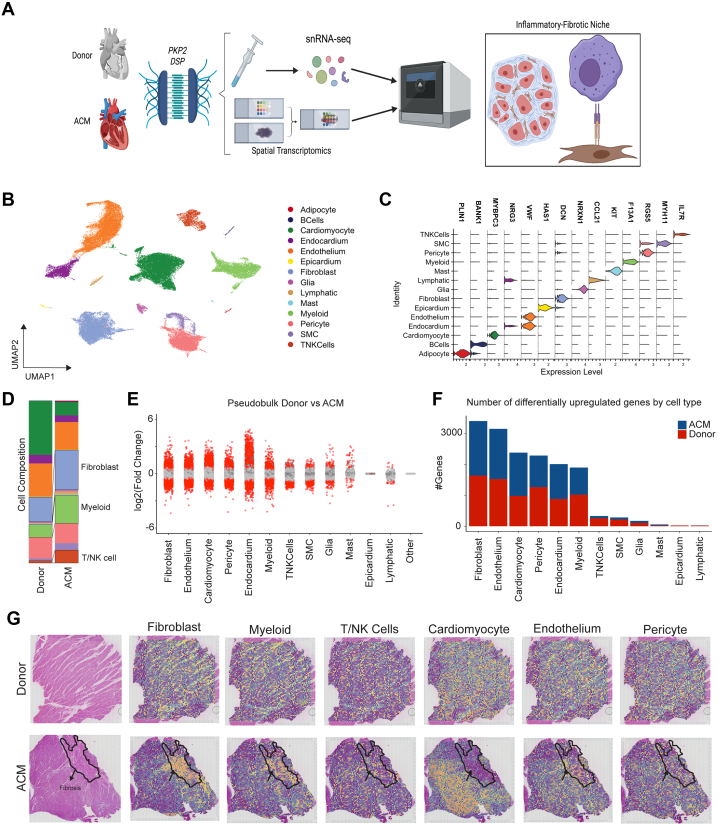
ACM Alters the Cardiac Cellular and Transcriptomic Environment (A) Study design schematic outlining human tissue sequencing methods. (B) Global uniform manifold approximation and projection (UMAP) with annotations of major cell populations. (C) Violin plot outlining major canonical markers identifying cell populations. (D) Composition plot displaying relative proportions of major cell types between donor and arrhythmogenic cardiomyopathy (ACM) samples. (E) Pseudobulk analysis displaying degree of gene expression changes across major cell types in donor vs ACM. (F) Number of total differentially up-regulated genes for each cell type. (G) Spatial transcriptomic plots displaying major cell populations overlaid on hematoxylin and eosin tissue images. NK = natural killer (cell); snRNAseq = single nuclei RNA sequencing.

We performed snRNAseq on transmural left ventricular specimens obtained from the apical anterior left ventricular wall of donor control subjects (n = 12) and patients with ACM (n = 6; including 3 patients with a *DSP* variant and 3 with a *PKP2* variant) (Supplemental Table 1) at the time of heart transplantation (Figure 1A). Following doublet removal and quality control (Supplemental Figure 1A), we performed dimensional reduction, uniform manifold approximation and projection (UMAP) construction, and differential gene expression to annotate cell types (Figure 1B). We identified 14 transcriptionally distinct cell types expressing canonical marker genes (Figure 1C). In addition, we constructed cell type–specific gene set scores and detected strong separation across clusters (Supplemental Figure 1B). Analysis of cell type composition demonstrated a robust expansion of fibroblast, myeloid, and T-cell populations in ACM myocardium compared to donor control subjects (Figure 1D). These expansions were observed across multiple individual ACM samples in our data set (Supplemental Figures 1C to 1F). To identify how ACM pathogenic variants perturb the transcriptional profile in a cell type–specific manner, we performed pseudobulk differential expression analysis at the patient level and tabulated up-regulated and down-regulated genes in ACM samples compared to donor control samples. We found fibroblasts, endothelial cells, cardiac myocytes, pericytes, endocardial cells, and myeloid cells harbored the most prominent transcriptional changes (Figures 1E and 1F). These data suggest that ACM is associated with remodeling of major cell types, including immune and stromal components of the myocardium.

### Spatial transcriptomics reveal formation of an inflammatory-fibrotic niche in ACM

To define the spatial organization of cell types enriched in ACM, we performed spatial transcriptomics sequencing of donor control (n = 2) and ACM (n = 3) left ventricular tissues. hematoxylin and eosin (H&E) staining showed areas of disrupted myocardial architecture and fibrosis, which we refer to as ACM lesions (Supplemental Figure 2). ACM specimens included those with a pathogenic *PKP2* (n = 2) and *DSP* (n = 1) variant (Figure 1A, Supplemental Tables 1 and 2). Each processed sample displayed high quality unique molecular identifier counts (Supplemental Figure 3).^20^^,^^21^ Because the spatial resolution of formalin-fixed, paraffin-embedded Visium technology is ∼40 μm^2^, each spot contains numerous cell types. To decipher the proportion of different cell types within each spot, we applied Tangram, which leverages our paired spatial transcriptomic and snRNAseq data sets to map cells into space.^26^ To visualize localization of major cell types, we plotted deconvolution scores for the major cell types overlaid on the H&E-stained image (Figure 1G). In donor hearts, cardiac myocytes represented the dominant cell population with homogenous organization of macrophages and fibroblasts. In ACM samples, we observed areas depleted of cardiac myocytes that were enriched with macrophages and fibroblasts, which corresponded with ACM lesions. Endothelial cells, pericytes, and T cells were homogenously distributed across the myocardium (Figure 1G).

**Figure 2:**
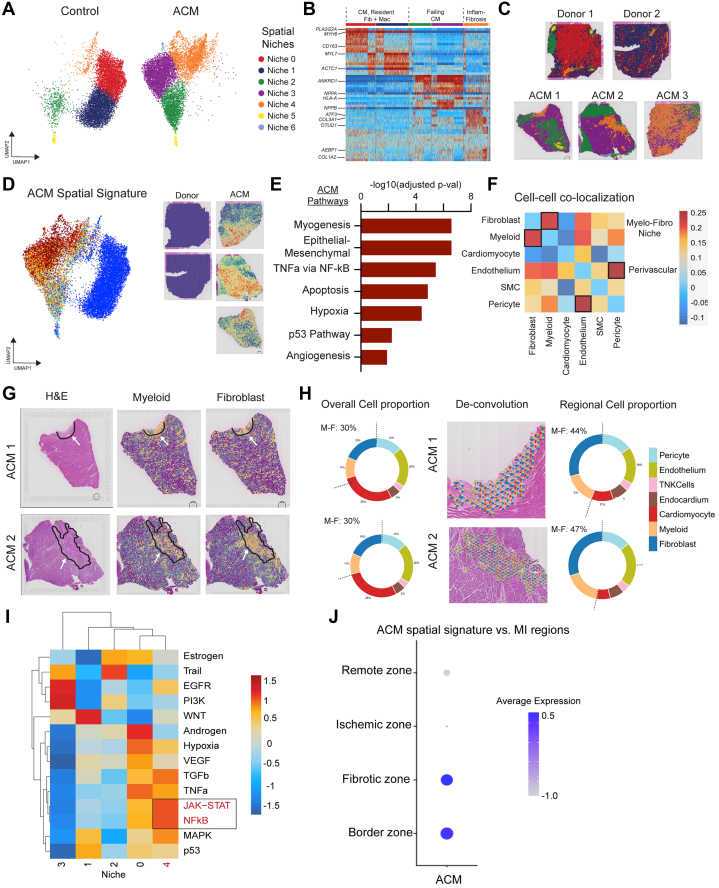
The Niche of ACM Includes Overlap Between Myeloid and Fibroblasts (A) Global UMAPs derived from spatial gene expression data outlining niches. (B) Heatmap displaying genes expressed by niches. (C) Niches overlaid onto tissue architecture to determine where niches are positioned in tissue space. (D) Top differentially expressed genes between ACM and donor control cells overlaid onto the spatial UMAP. (E) Pathway analysis displaying up-regulated pathways in ACM vs donor control cells. (F) Pearson correlation plot displaying likelihood that 2 cell types will be found in the same location in tissue. Darker blue indicates higher probability. (G) Hematoxylin and eosin (H&E) images from 2 ACM samples highlighting areas of damage, myeloid, and fibroblast concentration. (H) Circle graph displaying overall cell type proportions in tissue along with a region-specific cell type proportion in areas of tissue damage and fibrosis. (I) Heatmap displaying expression of major signaling pathways across niches. (J) Dot plot comparing ACM spatial signature against spatial signatures from the ischemic, remote, border, and fibrotic zones of myocardial infarction. CM = cardiomyocyte; Fib = fibroblast; Mac = macrophage; MI = myocardial infarct; NFκB = nuclear factor κB; SMC = smooth muscle cell; TNK= T/natural killer; TNF = tumor necrosis factor; other abbreviations as in Figure 1.

To further characterize the spatial architecture of ACM, we independently clustered the aggregated donor and ACM spatial transcriptomic data sets to identify unique spatial niches. Un-biased clustering identified 7 transcriptionally distinct spatial niches (Figure 2A). Niches 0 and 1 were enriched in donor compared to ACM samples and contained cardiac myocytes, macrophages expressing tissue resident markers, and fibroblasts. Niches 2 and 3 were enriched in ACM samples relative to donor control samples and contained cardiac myocytes expressing heart failure markers (*NPPA*, *NPPB*, and *ANKRD1*).^27^, ^28^, ^29^, ^30^ Niches 4, 5, and 6 were also increased in ACM samples relative to donor control samples and were composed of inflammatory macrophages and profibrotic fibroblasts (the latter referred to as the inflammatory-fibrotic niche) (Figures 2A and 2B) with niche 4 being the largest of the 3. We then coregistered the niche assignments onto the H&E-stained images and found that niche 4 was highly enriched in ACM lesions (Figure 2C).

To identify genes enriched in ACM-associated spatial niches, we performed a differential expression analysis between ACM and donor control samples and overlaid the signature onto the UMAP embedding (Figure 2D). This analysis revealed enrichment of the ACM signature in niches 3, 4, and 6 with strong colocalization to areas within and surrounding ACM lesions. Pathway enrichment analysis showed that the ACM transcriptional signature was enriched for myogenesis, epithelial-mesenchymal transition, tumor necrosis factor-α via NFκB, apoptosis, hypoxia, p53, and angiogenesis terms (Figure 2E).

To further characterize the ACM niches and identify which cell types colocalize in these spaces, we built a Pearson correlation coefficient matrix from tangram deconvolution scores and found that macrophages and fibroblasts form a myelo-fibro niche (a niche containing both myeloid cells and fibroblasts), whereas endothelial cells and pericytes form a perivascular niche (Figure 2F). We then overlaid tangram deconvolution scores for macrophages and fibroblasts in multiple ACM samples and saw strong colocalization of macrophage/fibroblast scores with areas of fibrosis (Figure 2G). To characterize relative cell abundance, we constructed aggregate pie charts across the entire tissue and found macrophages and fibroblasts were quantitatively expanded in ACM lesions (Figure 2H). To infer active signaling events in ACM lesions, we used PROGENy (https://saezlab.github.io/progeny/) for pathway analysis and discovered marked enrichment for NFκB and JAK-STAT signaling in niche 4 (the inflammatory-fibrotic niche) (Figure 2I). Given that our myocardial samples are from patients with end-stage heart disease, we sought to compare the ACM spatial signature we generated with the spatial signatures from various regions derived from human myocardial infarct (MI) samples published in a previous study.^31^ When comparing our ACM signature to the remote, ischemic, border, and fibrotic zones from MI samples, we observed a higher degree of overlap between the ACM signature and the border and fibrotic zones than the ischemic and remote zones (Figure 2J). These findings suggest some similarity between ACM disease and MI regions rich in macrophages and fibroblasts,^31^ but overall, they do not extend to MI as a whole.

### snRNAseq reveals expansion of POSTN expressing fibroblasts and inflammatory macrophages in ACM

**Figure 3:**
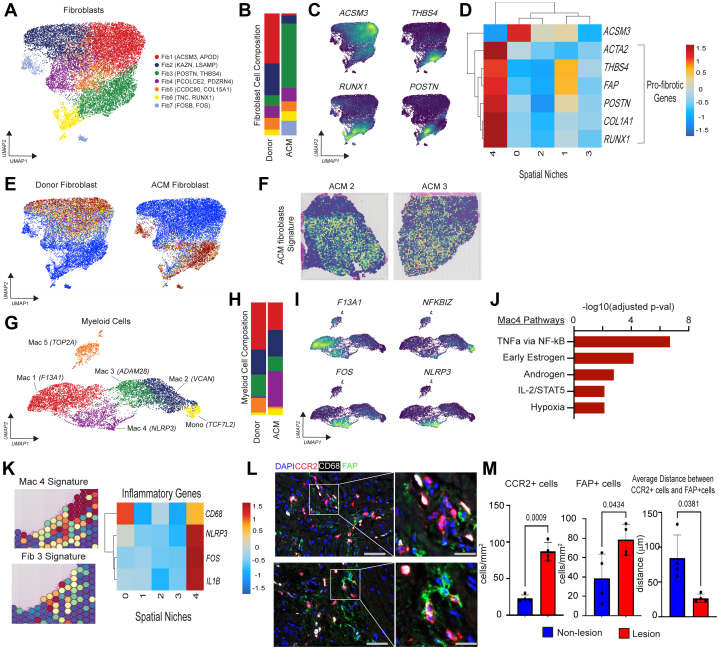
POSTN^+^ Fibroblasts and Inflammatory Macrophages Are Increased in ACM and Colocalize in Lesions (A) UMAP of fibroblast populations. (B) Composition plots of fibroblast populations between donor control and ACM cells. (C) Major fibroblast gene markers overlaid on the fibroblast UMAP. (D) Heatmap displaying expression of fibroblast markers across niches. (E) Fibroblast differential gene expression signature associated with either donor control or ACM cells overlaid onto the fibroblast UMAP space. (F) ACM fibroblast gene expression signature overlaid onto ACM tissue space. (G) UMAP of myeloid populations. (H) Composition plots of myeloid populations between donor control and ACM cells. (I) Major gene markers for myeloid populations overlaid on the myeloid UMAP. (J) Pathway analysis displaying pathways up-regulated in ACM vs donor control cells based on differentially expressed myeloid genes. (K) Colocalization of the inflammatory macrophage population (Mac4) and the POSTN^+^ fibroblast population (Fib3) in areas of tissue fibrosis and heatmap displaying expression of major inflammatory genes across spatial niches. (L) Immunofluorescence staining displaying colocalization of CCR2^+^ CD68^+^ macrophages and FAP^+^ fibroblasts in ACM tissue samples. Two independent samples were used. Broader images are captured at 20× magnification. Bars = 50 μm. (M) Quantification of CCR2^+^ macrophages, FAP^+^ fibroblasts, and average distance between the 2 cell types inside and outside ACM lesions (n = 4 for each group). Data are presented as mean ± SEM. Welch’s *t*-test was used for comparisons in (M). *P*-values inset. DAPI = 4ʹ,6-diamidino-2-phenylindole; other abbreviations as in Figures 1 and 2.

Given the enrichment of macrophages and fibroblasts within ACM lesions, we sought to characterize their precise cell states. To dissect the heterogeneity of fibroblasts and macrophages, we performed unbiased clustering of these populations. We identified 7 transcriptionally distinct fibroblast cell states (Fib1-7): Fib1 (*ACSM3, APOD*), Fib2 (*KAZN, LSAMP*), Fib3 (*POSTN, THBS4*), Fib4 (*PCOLCE2, PDZRN4*), Fib5 (*CCDC80, COL15A1*), Fib6 (*TNC, RUNX1*), and Fib7 (*FOSB, FOS*) (Figure 3A, Supplemental Figure 4A). Fib1 and Fib2 were enriched in donor control samples, whereas Fib3 and Fib7 were enriched in ACM samples (Figure 3B). This enrichment in Fib3 was present in multiple ACM samples (Supplemental Figures 5A and 5B). We calculated genes differentially expressed in donor control and ACM samples and visualized their expression using density plots. Genes with highest enrichment in donor control fibroblasts (*ACSM3*) were expressed in Fib1, whereas genes enriched in ACM (*THBS4, RUNX1*, and *POSTN*) were predominately expressed in Fib3 (Figure 3C). To identify spatial niches enriched with profibrotic cell states, we plotted *ACTA2*, *THBS4*, *FAP*, *POSTN*, *COL1A1,* and *RUNX1* (genes that have been previously implicated in tissue fibrosis and pathologic remodeling)^27^^,^^32^ across the spatial niches and found maximal enrichment in niche 4 (Figure 3D). Consistent with the analysis discussed, we performed pseudobulk differential gene expression in fibroblasts using our snRNAseq data and found that the donor control and ACM fibroblast signatures were enriched in Fib1-2 and Fib3, 6-7, respectively (Figure 3E). Moreover, the ACM fibroblast signature colocalized with niches 3 and 4, areas corresponding to and surrounding ACM lesions (Figure 3F).

We next sought to delineate macrophage states (Mac1-5) in ACM lesions. Unbiased clustering of myeloid cells within our snRNAseq data revealed 6 distinct cell states: monocytes (*TCF7L2*), Mac1 (*F13A1*), Mac2 (*VCAN*), Mac3 (*ADAM28*), Mac4 (*NLRP3*), and Mac5 (proliferating, *TOP2A*) (Figure 3G, Supplemental Figure 4B). Cell state composition analysis showed expansion of proliferating cells in donor control samples that is consistent with prior studies showing decreased myeloid proliferation in genetic dilated cardiomyopathy^27^^,^^33^^,^^34^ and expansion of *NLRP3*^+^ proinflammatory macrophages (Mac4) and monocytes in ACM (Figures 3G and 3H), consistent with a prior report.^35^ This enrichment in Mac4 was present in multiple ACM samples (Supplemental Figures 5C and 5D, Supplemental Table 2). Expression of *F13A1* localized to the resident macrophage population, whereas *FOS* and *NLRP3* were enriched in Mac4, and *NFKBIZ* was enriched in monocytes (Figure 3I). Pathway analysis of the marker genes for Mac4 show enrichment for tumor necrosis factor-α via NFκB, early estrogen, androgen, IL2/STAT5, and hypoxia activation (Figure 3J). These data highlight a key finding: that ACM inflammatory monocytes and macrophages show enriched NFκB-dependent signaling. We then plotted the gene signature of Mac4 and Fib3 in an ACM sample focusing on the regions of fibrosis and found a strong overlap between inflammatory macrophages and profibrotic fibroblasts, further highlighting the presence of an inflammatory-fibrotic niche (Figure 3K). Additionally, we plotted inflammatory genes such as *NLRP3, FOS,* and *IL1B,* which have previously shown to be enriched in inflammatory macrophages^16^, ^17^, ^18^^,^^27^^,^^32^^,^^36^ and found enrichment of these genes in spatial niche 4 (Figure 3K). Collectively, these findings support the idea that inflammatory macrophages and profibrotic fibroblast cell states are enriched in ACM lesions and may signal to one another, serving as a driving factor in the development of fibrosis in ACM, as demonstrated previously in myocardial infarction (Figure 3K).^31^^,^^32^ To validate predictions of cell composition within ACM lesions, we performed immunofluorescence staining for inflammatory macrophages (CD68^+^ and CCR2^+^) and activated fibroblasts (FAP^+^) and observed colocalization of both populations in ACM lesions (Figure 3L). Additionally, we observed that CCR2^+^ macrophages and FAP^+^ fibroblasts were present in greater numbers in ACM lesions compared to areas outside of these lesions and were located much closer together within lesions (Figure 3M). To determine whether this finding could be generalized to both ventricles, we performed H&E and immunofluorescence staining on right ventricular tissue from a subset of our human ACM samples. We identified similar ACM lesions and colocalization of inflammatory macrophages and activated fibroblasts in those lesions, suggesting that our finding is applicable to both ventricles in the setting of ACM disease (Supplemental Figure 6).

### snRNAseq of *Dsg2*^mut/mut^ mice reveals expansion of analogous pro-fibrotic fibroblasts and inflammatory macrophages

**Figure 4:**
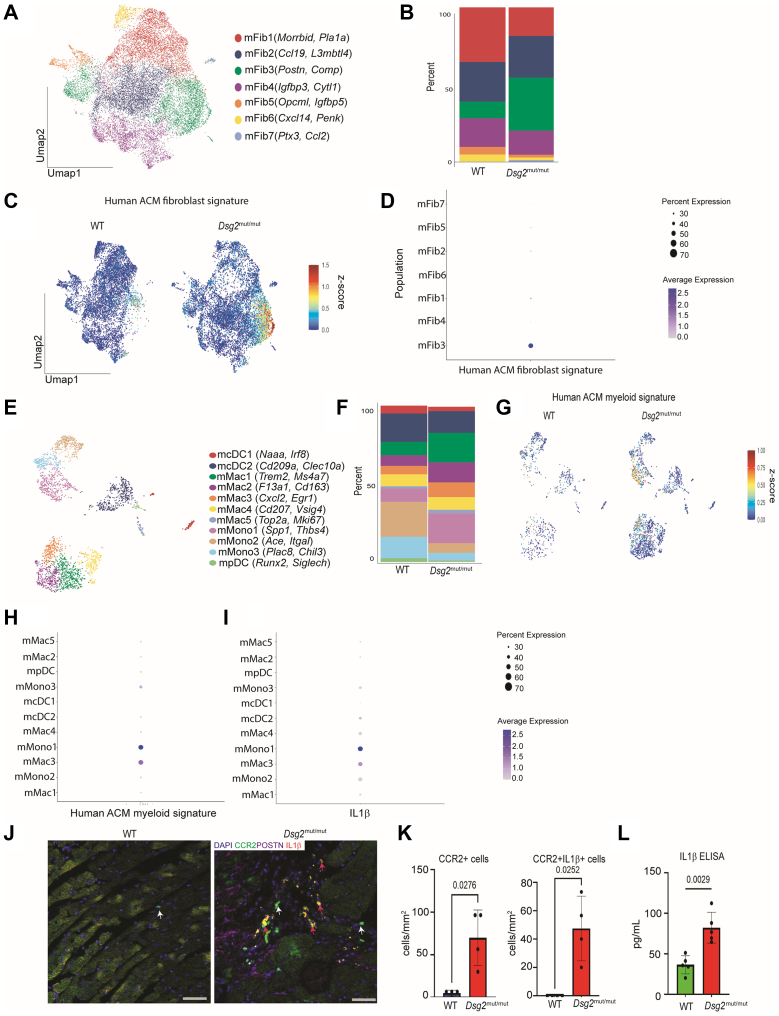
*Dsg2*^mut/mut^ Mice Have Analogous POSTN^+^ Fibroblast and Inflammatory Macrophage Populations as in Humans (A) Global UMAP of mouse fibroblast populations. (B) Composition plots comparing fibroblast populations between wild-type (WT) and *Dsg2*^mut/mut^ mice. (C) Gene expression score generated from mouse orthologs of differentially up-regulated human ACM fibroblast genes overlaid onto the mouse fibroblast UMAP. (D) Human gene expression score represented on a dot plot across mouse fibroblast populations. (E) Global UMAP of mouse myeloid populations. (F) Composition plots comparing myeloid populations between WT and *Dsg2*^mut/mut^ mice. (G) Gene expression score generated from mouse orthologs of differentially up-regulated human ACM myeloid genes overlaid onto the mouse myeloid UMAP. (H) Human gene expression score represented on a dot plot across mouse myeloid populations. (I) Expression score for *Il1b* measured across mouse myeloid populations. (J) Representative immunofluorescence staining images for CCR2, IL1B, and POSTN in WT and *Dsg2*^mut/mut^ hearts at 6 weeks of age. Images captured at 20× magnification. White arrows mark CCR2^+^ macrophages. Red arrows mark IL1B^+^ CCR2^+^ macrophages. Bar = 50 μm. (K) Quantification of CCR2^+^ cells and IL1B^+^ CCR2^+^ cells in WT and *Dsg2*^mut/mut^ hearts (n = 4 per group). Data are presented as mean ± SEM. (L) Quantification of IL1B enzyme-linked immunosorbent assay (ELISA) from heart homogenates from 6-week-old WT and *Dsg2*^mut/mut^ mice (n = 5 per group). Welch’s *t*-test was used for comparisons in (K) and (L). Data are presented as mean ± SEM. *P*-values inset. mcDC1 = myeloid cell dendritic cell state 1; mFib1 = murine fibroblast cluster 1; mMac1 = murine macrophage state 1; mMono1 = murine monocyte state 1; mpDC = murine plasmacytoid dendritic cell state; other abbreviations as in Figures 1 and 3.

To explore the contribution of macrophages and fibroblast populations in ACM pathogenesis, we used the homozygous *Dsg2* mutant (*Dsg2*^mut/mut^) mouse model of ACM. This strain recapitulates major pathologic and functional characteristics of ACM, including inflammation, fibrosis, impaired cardiac function, and arrhythmias.^14^^,^^15^^,^^19^ To determine whether an analogous macrophage-fibroblast axis contributed to disease in *Dsg2*^mut/mut^ mice, we performed targeted single-cell RNA sequencing of fibroblast and myeloid populations in 6-week-old *Dsg2*^mut/mut^ mice and age-matched WT hearts (Supplemental Figure 7A). After performing quality control (Supplemental Figure 7B), murine fibroblast clustering identified 7 transcriptionally different clusters (mFib1-7): mFib1 (*Morrbid, Pla1a)*, mFib2 (*Ccl19, L3mbtl4*), mFib3 (*Postn, Comp*), mFib4 (*Igfbp3, Cytl1*), mFib5 (*Opcml, Igfbp5*), mFib6 (*Cxcl14, Penk)*, and mFib7 (*Ptx3, Ccl2*) (Figure 4A, Supplemental Figure 7C). mFib3 and mFib7 were enriched in *Dsg2*^mut/mut^ mice, whereas mFib1, mFib5, and mFib6 were enriched in WT mice (Figure 4B). To determine whether the *Postn* enriched cluster in mice (mFib2) was similar to the *POSTN* enriched cluster in humans (Figure 3), we generated a gene set signature score using the human genetic expression data (converted into mouse orthologs) (Supplemental Table 3) and overlaid that score onto the mouse UMAP (Figure 4C). The ACM human fibroblast gene signature was robustly expressed by the mFib3 cluster, suggesting the existence of a transcriptionally analogous population of fibroblasts present in *Dsg2*^mut/mut^ mice (Figure 4D).

Further analysis of the myeloid cell populations yielded 12 transcriptionally unique states: myeloid cell dendritic cell states: mcDC1 (*Irf8, Naaa*), mcDC2 (*Cd209a, Clec10a*); murine macrophage states: mMac1 (*Trem2, Ms4a7*), mMac2 (*F13a1, Cd163*), mMac3 (*Cxcl2, Egr1*), mMac4 (*Cd207, Vsig4*), mMac5 (*Top2a, Mki67*); murine monocyte states: mMono1 (*Spp1, Thbs4*), mMono2 (*Ace, Itgal*), mMono3 (*Plac8, Chil3*); and murine plasmacytoid dendritic cell state: mpDC (*Runx2, Siglech*) (Figure 4E, Supplemental Figure 7D). mMono1, mMac1, and mMac3 were significantly overrepresented in *Dsg2*^mut/mut^ mice compared to WT mice, whereas mMono2 and mMono3 were enriched in WT mice (Figure 4F). Similar to the fibroblasts, we generated a gene set signature score using the human expression data (Supplemental Table 4) and overlaid it onto the mouse UMAP to determine whether there was an analogous population of inflammatory macrophages in *Dsg2*^mut/mut^ mouse hearts (Figure 4G). The ACM human myeloid signature was robustly expressed by mMono1 and mMac3 (Figure 4H), 2 populations that have previously been shown to be derived from CCR2^+^ monocytes.^37^ Subsequent analysis revealed that these populations also robustly express *Il1b* (Figure 4I), suggesting that these are an analogous population of inflammatory macrophages like those found in hearts from patients with ACM. To further validate these findings, we performed immunofluorescence staining for CCR2, IL1B, and POSTN in 6-week-old WT and *Dsg2*^mut/mut^ mouse hearts. We observed increased numbers of CCR2^+^ macrophages expressing IL1B in *Dsg2*^mut/mut^ mouse hearts compared to WT control hearts (Figures 4J and 4K). Additionally, we performed IL1B enzyme-linked immunosorbent assays from heart homogenates from 6-week-old WT and *Dsg2*^mut/mut^ mice and observed increased IL1B levels in *Dsg2*^mut/mut^ mice compared to WT control mice (Figure 4L), indicating early immune activation in the absence of cardiac dysfunction. Overall, these findings point to the presence of a similar inflammatory-fibroblast axis in *Dsg2*^mut/mut^ mouse hearts, suggesting that this mouse model mirrors human ACM disease at the cellular and transcriptional level.

### Targeting IL1B attenuates disease characteristics in *Dsg2*^mut/mut^ mice

**Figure 5:**
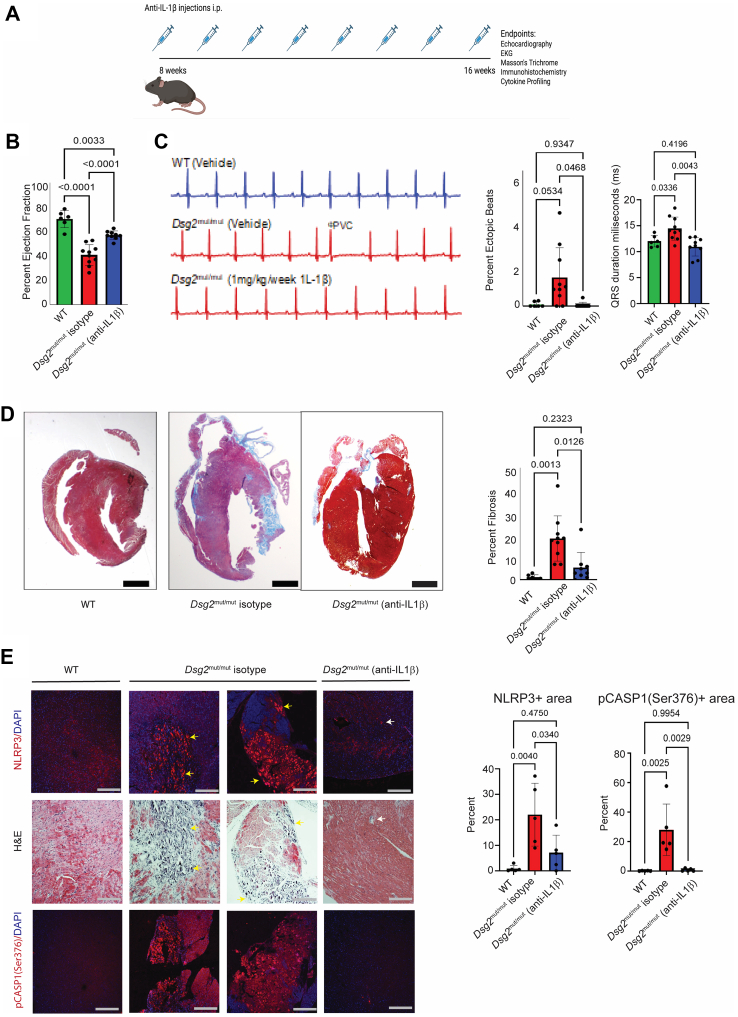
IL1B Blockade Significantly Attenuates Disease in *Dsg2*^mut/mut^ Mice (A) Study design outlining treatment schedule of neutralizing IL1B antibody. (B) Percentage of ejection fraction (n = 6, 10, and 9 for each group, respectively). (C) Representative electrocardiograms from each cohort and quantification of the proportion of ectopic beats and QRS duration (n = 6, 10, and9 for each group, respectively). (D) Representative trichrome images from each cohort and quantification of fibrosis percentage (n = 6, 10, and 9 for each group, respectively). Bars = 1 mm. (E) Serial sections of representative immunostained hearts probed for NLRP3, H&E, and phospho-(p)CASP1 (serine 376): n ≥ 5 hearts/cohort/stain; yellow arrows, NLRP3^+^ staining localized in areas of myocardial lesions that additionally corresponds with serial sections in H&E stains; white arrowhead, NLRP3^+^ staining localized around vessel lumen. Bars = 200 μm. Quantification of NLRP3^+^ area and pCASP1^+^ area (n = 5 per group). Brown-Forsythe and Welch analysis of variance testing used for graphs (B to E). Data are represented as mean ± SEM. *P*-values inset.

IL1B is a primordial inflammatory cytokine of the innate immune response, produced predominantly by macrophages.^32^ Recent studies,^38^, ^39^, ^40^, ^41^ including the CANTOS (Canakinumab Anti-inflammatory Thrombosis Outcomes Study) trial and VCU-ART (Virginia Commonwealth University Anakinra Remodeling Trial),^40^ have explored the efficacy of IL1B blockade in various forms of cardiovascular disease, including myocardial infarction and atherosclerosis. Yet, the role of targeting IL1 signaling in ACM is poorly understood. Given our findings of increased *Il1b*-expressing inflammatory macrophages in *Dsg2*^mut/mut^ mouse hearts and enrichment for *NLRP3* in corresponding human macrophages, we set out to determine whether IL1B blockade can mitigate ACM disease progression prior to overt cardiac remodeling and fibrosis. Therefore, 8-week-old WT and *Dsg2*^mut/mut^ mice were treated with either isotype control (10 mg/kg/wk of mouse anti-IgG antibody) or a mouse anti-IL1B neutralizing antibody (1 mg/kg/wk) with similarity to canakinumab once a week for 8 weeks (Figure 5A). Prior to and following treatment, we performed echocardiography and found a significant improvement in left ventricular ejection fraction (Figure 5B) in *Dsg2*^mut/mut^ mice treated with anti-IL1B antibody compared to those that received isotype control. Similarly, we observed a decrease in the frequency of premature ventricular contractions and reduced ventricular ectopy (QRSd) in anti-IL1B antibody treated *Dsg2*^mut/mut^ mice compared to isotype control mice (Figure 5C). On harvesting hearts from these cohorts, we assessed myocardial fibrosis and found a significant decrease in fibrotic area in anti-IL1B antibody treated *Dsg2*^mut/mut^ mice relative to isotype-treated counterparts (Figure 5D). Additionally, we performed multiplex cytokine array analysis and found that anti-IL1B antibody treatment decreased the levels of several proinflammatory and profibrotic cytokines, including CD14, CXCL2, CXCL9, interferon-γ, osteopontin, and POSTN (Supplemental Table 5).

The NLRP3 inflammasome, a cytosolic complex found in proinflammatory immune cells, has been implicated in several cardiovascular diseases and is responsible for the processing and release of IL1B using active CASP1.^35^^,^^42^ Given our results demonstrating the expansion of *NLRP3*^+^ macrophages and monocytes in human ACM hearts, we evaluated myocardial tissue for NLRP3 and phosphorylated CASP1 in *Dsg2*^mut/mut^ and WT mice. In hearts from isotype-treated *Dsg2*^mut/mut^ mice, NLRP3 and phosphorylated CASP1 were exclusively localized in myocardial lesions that additionally showed extensive infiltrating immune cells, a finding not observed in anti-IL1B antibody treated *Dsg2*^mut/mut^ mice (Figure 5E).

To assess whether a similar benefit could be derived from anti-IL1B antibody treatment at a time point in which cardiac dysfunction and biventricular fibrosis are quite evident,^14^^,^^15^^,^^19^ we treated WT or *Dsg2*^mut/mut^ mice from 16- to 24-weeks of age (Supplemental Figure 8A). At this point of intervention, we found modest improvements in cardiac function in anti-IL1B antibody–treated *Dsg2*^mut/mut^ mice, whereas cardiac function deteriorated further in mice that received isotype control (Supplemental Figure 8B). The modest recovery of contractile function in anti-IL1B antibody–treated mice was associated with a substantial reduction in myocardial fibrosis and ectopic beats (Supplemental Figures 8C and 8D). The more robust improvement in ACM disease at an earlier time point suggests that whereas anti-IL1B treatment may have the greatest effect during the early inflammatory stage of disease prior to overt fibrotic remodeling,^15^ some benefit can still be achieved at later time points. Overall, these findings indicate that therapies targeting inflammation in ACM can provide substantial reductions in disease burden across the natural history of disease.

### Treatment of *Dsg2*^mut/mut^ mice with an anti-IL1B antibody alters the cardiac transcriptional environment

**Figure 6:**
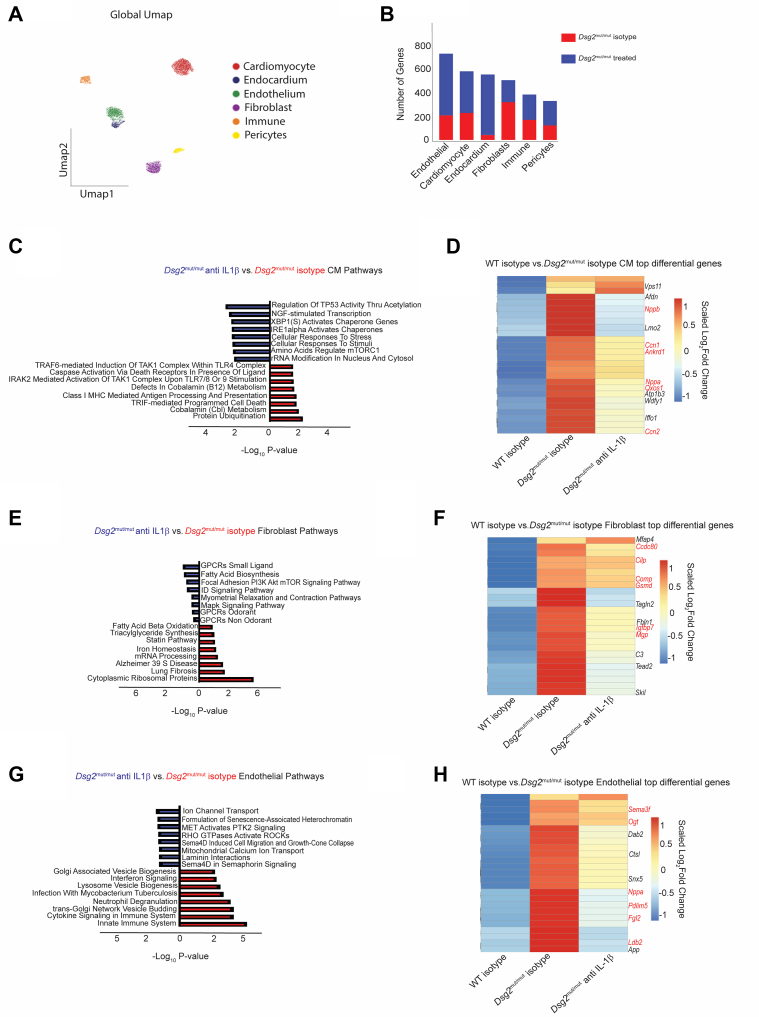
Early IL1B Blockade Alters the Transcriptomic Environment in *Dsg2*^mut/mut^ Mice (A) Global UMAP of cell populations captured in iCell8cx sequencing. (B) Total number of differentially expressed genes between *Dsg2*^mut/mut^ isotype control and *Dsg2*^mut/mut^ anti-IL1B–treated mice across different cell types. (C) Pathway analysis displaying differentially expressed pathways in cardiac myocytes between *Dsg2*^mut/mut^ isotype control and *Dsg2*^mut/mut^ anti-IL1B–treated mice. (D) Heatmap displaying top differentially expressed cardiac myocyte genes between WT isotype control and *Dsg2*^mut/mut^ isotype control mice and compared to those same genes in *Dsg2*^mut/mut^ anti-IL1B–treated mice. (E) Pathway analysis displaying differentially expressed pathways in fibroblasts between *Dsg2*^mut/mut^ isotype control and *Dsg2*^mut/mut^ anti-IL1B–treated mice. (F) Heatmap displaying top differentially expressed fibroblast genes between WT isotype control and *Dsg2*^mut/mut^ isotype control mice and compared to those same genes in *Dsg2*^mut/mut^ anti-IL1B–treated mice. (G) Pathway analysis displaying differentially expressed pathways in endothelial cells between *Dsg2*^mut/mut^ isotype control and *Dsg2*^mut/mut^ anti-IL1B– treated mice. (H) Heatmap displaying top differentially expressed endothelial genes between WT isotype control and *Dsg2*^mut/mut^ isotype control mice and compared to those same genes in *Dsg2*^mut/mut^ anti-IL1B–treated mice. GPCR = G-protein-coupled receptor; GPTase = guanosine triphosphatase; MHC = major histocompatibility complex; mRNA = messenger RNA; other abbreviations as in Figure 1, Figure 2, and 4.

To delineate the transcriptional changes that occur following IL1B neutralization in *Dsg2*^mut/mut^ mice, we performed snRNAseq on 16-week-old WT and *Dsg2*^mut/mut^ mice following 8 weeks of either isotype control or anti-IL1B antibody treatment. We used the iCell8cx SMART-seq Pro platform (Takara Bio), which allows for the capture of full-length complementary DNA and a greater number of genes per nuclei (>5,000) compared to other sequencing technologies.^43^ After performing quality control (Supplemental Figure 9A), we identified 6 transcriptionally distinct cell types (Figure 6A) marked by major canonically expressed genes (Supplemental Figure 9B). We observed that whereas the major composition of cell types did not change following anti-IL1B antibody treatment in *Dsg2*^mut/mut^ mice (Supplemental Figure 9C), there was a large number of differentially expressed genes between the anti-IL1B antibody and isotype-treated *Dsg2*^mut/mut^ cohorts in all cell types (Figure 6B). The largest number of differentially expressed genes—as well as—captured nuclei were in endothelial cells, cardiac myocytes, and fibroblasts. To further investigate changes in the cardiac myocyte gene expression, we performed pathway analysis using the top 25 differentially expressed genes between anti-IL1B antibody– and isotype-treated *Dsg2*^mut/mut^ hearts. We observed an up-regulation in pathways associated with NFκB-mediated inflammation and cell death in isotype-treated *Dsg2*^mut/mut^ mice, whereas anti-IL1B antibody–treated cardiac myocytes displayed enrichment in pathways associated with homeostasis and stress response (Figure 6C). These findings are consistent with our previously published observations indicating that NFκB activity in cardiac myocytes participates in myocardial cell death, cardiac inflammation, arrhythmias, and reduced contractile function.^15^

Next, we determined whether the broader ACM phenotype had been reversed in cardiac myocytes following anti-IL1B antibody treatment. To address this question, we determined the expression values of the top 25 differentially expressed genes between WT and *Dsg2*^mut/mut^ mouse hearts and assessed whether these genes were affected by anti-IL1B antibody treatment in *Dsg2*^mut/mut^ hearts (Figure 6D). We observed that a variety of genes up-regulated in ACM cardiac myocytes were down-regulated following anti-IL1B antibody treatment, including major heart failure and inflammation associated genes (highlighted in red, Figure 6D).^44^, ^45^, ^46^

We applied the same analysis to the fibroblast and endothelial cell populations and observed an up-regulation in pathways associated with fibrosis in isotype-treated *Dsg2*^mut/mut^ hearts, whereas the anti-IL1B antibody–treated fibroblasts displayed enrichment in pathways associated with homeostatic signaling and contraction (Figure 6E). As for cardiac myocytes, many of the major gene signatures up-regulated in *Dsg2*^mut/mut^ hearts relative to WT were down-regulated following treatment, including a number of genes associated with cardiac disease and fibrosis (Figure 6F).^47^, ^48^, ^49^, ^50^, ^51^ In endothelial cells, we observed an up-regulation in pathways associated with innate immune signaling and inflammation in isotype-treated *Dsg2*^mut/mut^ mice, whereas anti-IL1B antibody–treated endothelial cells displayed enrichment in pathways associated with ion transport and guanosine triphosphatase signaling (Figure 6G). Again, the majority of genes up-regulated in *Dsg2*^mut/mut^ hearts relative to WT were down-regulated following treatment including a number associated with endothelial stress and dysfunction (Figure 6H).^52^, ^53^, ^54^, ^55^, ^56^, ^57^

### NFκB nuclear localization, myocardial infiltration of CCR2/CD68^+^ macrophages, and POSTN activation is reduced in anti-IL1B antibody–treated *Dsg2*^mut/mut^ mice

**Figure 7:**
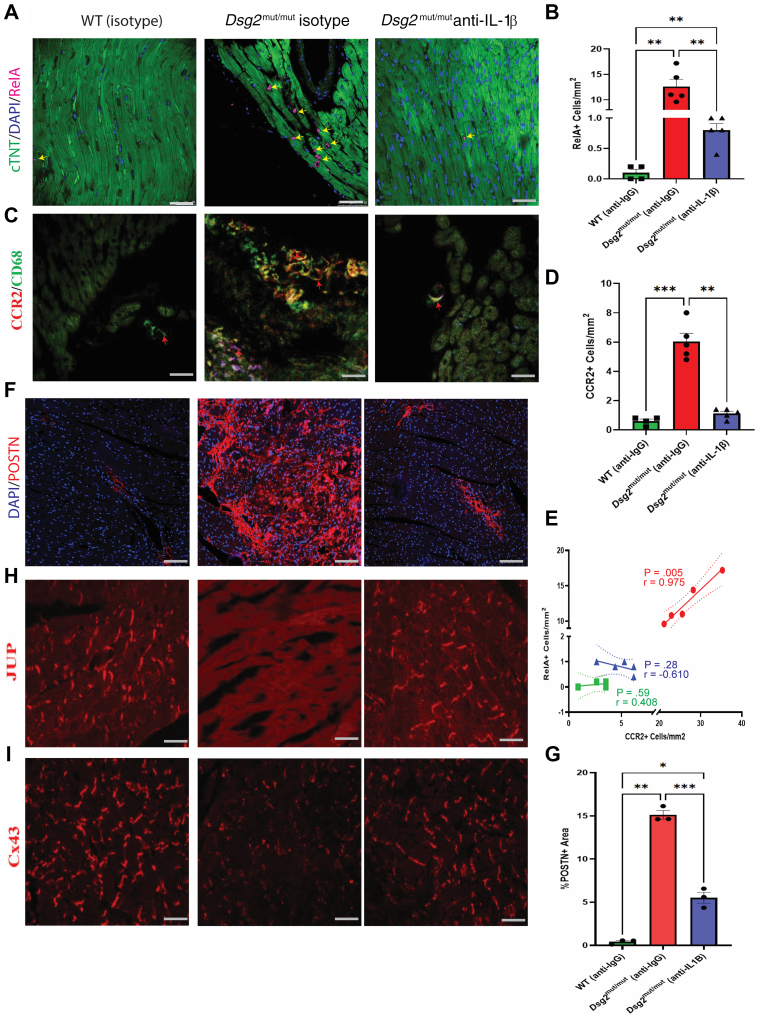
IL1B Neutralization Prevents NFκB Nuclear Localization and Reduces Infiltrating Myocardial CCR2/CD68^+^ Macrophages and POSTN Activation in *Dsg2*^mut/mut^ Mice (A,C,F,H,I) Representative immunostained hearts probed for RelA, CCR2/CD68, POSTN, JUP, and connexin-43 (Cx43): n ≥ 3 hearts/cohort/stain; black arrows, RelA+ nuclei; red arrowheads, CCR2/CD68^+^ macrophages; bars = 50 μm. (B) Number of cells per square millimeters positive for nuclear RelA localization. (D) Number of cells per square millimeters positive for CCR2. (E) Pearson correlation analysis for cells that showed dual labeling for RelA and CCR2 (*P* values and *r* values inset). Data presented as slope (solid line) and 95% CI (dotted lines). (G) Percentage of area that is POSTN^+^ (n = 3 per group). (B,D,G) Data are mean ± SEM. ∗*P* < 0.05, ∗∗*P* < 0.01, and ∗∗∗*P* < 0.001 using 1-way analysis of variance with Tukey post hoc analysis. cTnT = cardiac troponin T; IgG = immunoglobulin G; other abbreviations as in Figure 2, Figure 3, Figure 4.

Given our findings from the human spatial transcriptomics data that demonstrated areas of myocardial loss harbored macrophages expressing NFκB-dependent transcripts as well as our findings from the iCell8cx data displaying a reduction in pathways associated with NFκB activation in cardiac myocytes, we aimed to determine whether NFκB nuclear localization in myocardial cells was decreased in anti-IL1B–treated ACM mice. We observed robust immunoperoxidase signal for RelA/p65 in in isotype-treated *Dsg2*^mut/mut^ hearts, a finding not observed in WT or anti-IL1B–treated *Dsg2*^mut/mut^ myocardium (Figures 7A and 7B). Additionally, we previously showed that NFκB signaling in cardiac myocytes is liable for mobilizing CCR2^+^ cells to ACM hearts, where they promote myocardial injury and arrhythmias.^15^ Accordingly, we assessed the number of infiltrating macrophages via double immunolabeling for CCR2 and CD68 (Figures 7C and 7D). There was a strong, positive correlation between cells that demonstrated immunoreactivity for both CCR2 and CD68 and those that expressed RelA/p65 in isotype-treated *Dsg2*^mut/mut^ hearts, which was absent in the hearts of anti-IL1B–treated *Dsg2*^mut/mut^ mice (Figure 7E). These findings indicate infiltrating CCR2^+^/CD68^+^ macrophages in ACM hearts can be blocked via anti-IL1B treatment. To further validate our transcriptomic data, we aimed to determine whether pathogenic fibrosis and tissue remodeling was reduced following anti-IL1B treatment by staining for POSTN. We observed a marked reduction in myocardial POSTN deposition in anti-IL1B–treated *Dsg2*^mut/mut^ mice compared to those treated with isotype control (Figures 7F and 7G), suggesting a significant reduction in pathogenic fibrosis following anti-IL1B treatment.

Loss of JUP and connexin-43 (Cx43) at the myocyte-myocyte intercalated disc are classical pathologic hallmarks of ACM.^58^ Additionally, recent works reflect that IL1B released^59^ from both immune cells and myofibroblasts^60^ resulted in reduced Cx43 expression and gap junction localization. Therefore, we determined whether the loss of Cx43 at the intercalated disc in *Dsg2*^mut/mut^ hearts^14^ could be prevented by anti-IL1B antibody. Depressed cardiac myocyte junctional immunoreactive signal for JUP and Cx43 was noted in all *Dsg2*^mut/mut^ mice treated with isotype control, compared to WT myocardium (Figures 7H and 7I). Abnormal distributions for both proteins were fully corrected in hearts from *Dsg2*^mut/mut^ mice treated with anti-IL1B antibody (Figures 7H and 7I).

## Discussion

Inflammation and fibrosis are long-recognized features of ACM. Postmortem explants have revealed significant myocardial fibrosis and inflammatory infiltrates within both the right and left ventricular walls. Moreover, serum levels of inflammatory cytokines, including IL1B and C-reactive protein, are elevated in ACM patients.^2^^,^^11^^,^^13^^,^^61^ Additionally, there has been greater recognition that some individuals diagnosed with acute or recurrent myocarditis carry aggressive ACM mutations, most notably *DSP* variants.^62^ Recent work employing established mouse models of ACM has provided crucial insights implicating the contributions of inflammation and monocyte recruitment in ACM pathogenesis.^15^^,^^63^, ^64^, ^65^ Despite these advances, the emerging field of cardioimmunology has an incomplete understanding of the immune and stromal landscape of ACM and little is known regarding effector mechanisms that drive myocardial inflammation.

Here we used snRNAseq and spatial transcriptomics to define the cellular landscape of human ACM. We uncovered robust expansion of the myeloid and fibroblast populations that localize with ACM lesions. These cell populations harbor the greatest number of differentially expressed genes between donor and ACM hearts. Detailed characterization of fibroblast and myeloid populations expanded in ACM myocardium revealed specific increases in *POSTN*-expressing fibroblasts and *NLRP3* expressing inflammatory macrophages. Additionally, we observed a decrease in *F13A1* expressing resident macrophages in ACM hearts compared to donor control hearts. Similar shifts in fibroblast and macrophage states have been previously reported in myocardial infarction^32^ and some forms of dilated cardiomyopathy.^27^^,^^33^^,^^34^ Our results are consistent with and expand on past ACM sequencing studies.^35^

Using spatial transcriptomics, we uncovered the spatial organization of cell types that reside within areas of tissue damage (referred to as ACM lesions). Our data indicated that cardiac myocytes were lost within ACM lesions, which were instead predominantly composed of fibroblasts and macrophages. These lesions were surrounded by niches that contained cardiac myocytes that expressed markers typically observed in heart failure.^27^, ^28^, ^29^, ^30^ ACM lesions resembled an immune-fibrotic niche, which largely comprised *POSTN* fibroblasts and inflammatory macrophages. This niche displayed enrichment for inflammatory and fibrotic pathways including NFκB, tumor necrosis factor-α, and TGFB. Of note, this niche possessed similarities to what is observed in the fibrotic and border zones of myocardial infarctions,^31^^,^^32^ highlighting the potential importance of necroinflammation in ACM. Whereas there is a degree of overlap between the spatial signatures of ACM and the fibrotic and border zones of myocardial infarction, there is not a 1:1 correspondence, suggesting that though not entirely unique, the ACM signature is likely a product of the underlying genetic disease rather than just end-stage heart failure findings alone.

A key component of inflammatory-related processes is crosstalk between immune cells and stromal elements within the tissue microenvironment. Given the proximity of inflammatory macrophages and fibroblasts within ACM lesions, the direct paracrine communication between these cell types may contribute to disease pathogenesis of ACM. To explore a causative relationship between such effector and signaling mechanisms, it is imperative to identify robust models that recapitulate the human disease and are experimentally tractable.

Various in vitro and in vivo models of ACM have been developed over the years^66^ that primarily include models harboring analogous pathogenic gene variants seen in patients with ACM. We leveraged the *Dsg2*^mut/mut^ mouse model, which recapitulates several key pathologic and functional aspects of ACM, and examined whether analogous macrophage and fibroblast populations exist in this ACM model. Indeed, single-cell RNA sequencing of the myeloid and fibroblast populations revealed increases in transcriptionally similar *Postn*^+^ fibroblasts and inflammatory monocyte and macrophage subsets. These populations expressed many of the genes enriched in human ACM, suggesting that the *Dsg2*^mut/mut^ mouse model could serve as a platform to dissect inflammatory mechanisms that are relevant to human ACM pathogenesis. It is interesting to note that our human sequencing data were derived from patients with *PKP2* and *DSP* variants, which highlights the substantial overlap between gene variants belonging to the cardiac desmosome. Additionally, the analogous lesions, inflammatory macrophages, and activated fibroblasts identified in human ACM were present in *Dsg2*^mut/mut^ mice as early as 6 weeks of age (prior to the development of major cardiac dysfunction), further supporting that these characteristics are a product of ACM pathogenesis and not just end-stage heart disease.

IL1B is a potent inflammatory cytokine elevated in various forms of cardiac disease and studied in previous clinical trials.^38^, ^39^, ^40^^,^^67^ IL1B plays an important role in adverse remodeling following cardiac injury and promotes myocardial fibrosis via a paracrine communication axis between inflammatory macrophages and fibrotic fibroblasts.^32^ Although patients with ACM display elevated serum levels of IL1B,^2^^,^^11^^,^^13^ its role in ACM pathogenesis is undetermined. Our sequencing data in both human and mouse revealed an increase in NLRP3- and IL1B-expressing macrophages in ACM. Together these findings suggest that IL1B may be a useful therapeutic target in ACM. Blockade of IL1B at 8 weeks of age in *Dsg2*^mut/mut^ mice using an anti-IL1B neutralizing antibody, resulted in remarkable improvement in contractile function, decreased fibrosis, and diminished frequency of premature ventricular depolarizations. These findings align with a previously reported study that used an NLRP3 inhibitor in a mouse model of ACM.^35^

To determine whether targeting IL1B might be beneficial in advanced disease, we also blocked IL1B in 16-week-old *Dsg2*^mut/mut^ mice. We observed modest improvements in ejection fraction and a substantial decrease in cardiac fibrosis. These findings suggest benefits across the natural history of ACM with greater efficacy at earlier time points, which are hypothesized to resemble periods of greater inflammation.^15^ In mouse models, monocytes appear to peak at 4-6 weeks of age.^15^ Nevertheless, there is a large degree of heterogeneity in the progression of ACM, especially in humans.^1^ Imaging cardiac inflammation using novel positron emission tomography tracers may be a useful strategy for identifying which patients would benefit most from anti-inflammatory intervention.^68^^,^^69^

To uncover potential transcriptional changes in the cardiac environment following treatment with anti-IL1B antibody, we performed snRNAseq on 16-week-old WT and *Dsg2*^mut/mut^ treated hearts using the iCell8cx SMART-seq Pro platform to leverage its ability to obtain more genes per nuclei. Analysis of the sequencing results revealed a plethora of differentially expressed genes. Within cardiac myocytes, we observed substantial reductions in the ACM cardiac gene signature in anti-IL1B antibody–treated ACM mutants. Using pathway analysis, we observed down-regulation of NFκB-induced inflammation and cell death–associated pathways in following IL1B antibody–treated ACM mutants. We have previously demonstrated that NFκB-dependent cell death in cardiac myocytes is a major determinant regulating the recruitment of inflammatory monocytes in ACM hearts via transcriptional up-regulation of potent chemotactic molecules.^15^ IL1B blockade has been shown to reduce cardiac fibrosis,^70^ intestinal cell death in a model of small intestine enteropathy,^71^ and βislet cell death in a rat model of type I diabetes.^72^ The reduction of cardiac myocyte cell death, fibrosis, and inflammation highlights a probable mechanism of action by which IL1B blockade attenuates ACM pathogenesis.

### Study limitations

The human myocardial samples studied were obtained from patients at time of heart transplantation; thus, these hearts were collected during advanced stages of disease progression. As a result, we were unable to distinguish the cellular and transcriptional landscape in ACM hearts during different stages of disease (ie, “concealed” vs “hot” phases) at time of sequencing. Given the heterogenous disease course and variable age of diagnosis, this may have an impact on the cellular populations and transcriptional states we detected. That being said, the major characteristic ACM lesions containing inflammatory macrophages and activated fibroblasts were found in our mouse model of disease as early as 6 weeks of age (prior to the development of advanced cardiac dysfunction), suggesting that this finding develops early in ACM pathogenesis.

Whereas we did not have patient cardiac magnetic resonance data from each heart explant we obtained, each patient underwent exome sequencing and only those with a mutation in a single ACM causative gene were selected for our transcriptomic analysis.

Although patient samples were obtained from regions of myocardial injury, prior works have demonstrated that both innate immune activation in cardiac myocytes and immune cell infiltrates occur in both ventricles even when macroscopic disease is confined to the right ventricle.^73^ Additionally, in vitro studies that used neonatal rat ventricular myocytes expressing a *JUP*^2157del2^ transgene and/or induced pluripotent stem cell–derived cardiomyocytes harboring *DSG2* and *PKP2* pathogenic variants all showed robust cardiomyocyte pRelA nuclear localization (ie, NFκB activation).^19^^,^^74^ Thus, these prior findings indicate that immune activation in desmosomal-linked ACM can occur in ventricles with no evidence of myocardial injury and in the absence of infiltrating immune cells or exogenous inflammatory stimuli (ie, cell autonomous).

Additionally, whereas we sequenced hearts harboring 2 of the most prevalent genes that give rise to ACM, we recognize that additional pathogenic variants in several other genes are associated with an ACM-like phenotype.^7^ Although our data suggest that there is some degree of conservation across desmosomal genes, it is possible that pathogenic variants in structural or Z-disc genes may display distinct features. Additionally, we acknowledge that the clinical course and specific pathology of ACM can vary substantially across different ACM mutations and that this heterogeneity is an important characteristic of the disease. Nevertheless, we still believe there is tremendous value in discovering unifying pathologic mechanisms that may underlie and contribute to disease pathogenesis in various ACM mutational etiologies because these may lend insight into diagnostic or therapeutic approaches that could benefit all patients with ACM. We also recognize that our myocardial samples come entirely from the left ventricle of ACM patients. Whereas the lesions containing inflammatory macrophages and activated fibroblasts were also present in the right ventricles of ACM patients, suggesting a biventricular phenomenon, adipocyte dysplasia, a major clinical finding in ACM^1^^,^^2^^,^^6^ is observed primarily in the right ventricle,^1^^,^^2^^,^^6^^,^^75^ therefore a more in-depth analysis focused on right ventricular myocardial samples would be fruitful in understanding the specific contribution from adipocytes to ACM pathogenesis. Lastly, whereas we focus on the role of IL1B, it is likely that other inflammatory signaling pathways contribute to ACM.

## Conclusions

We observe a conserved expansion of inflammatory macrophages and fibrotic fibroblasts in ACM in both mice and humans. These cell types are spatially localized to ACM-marked areas of tissue damage and fibrosis (ie, lesions). We demonstrate that IL1B produced by proinflammatory macrophages participates in ACM pathogenesis by driving inflammation, fibrosis, contractile dysfunction, and an arrhythmogenic substrate. Our findings highlight the utility of anti-inflammatory therapies for ACM, which may serve as a new avenue to treat this devastating disease.

### Data availability

Raw and processed sequencing files will be uploaded to the Gene Expression Omnibus. Code is available on request to the authors.

Perspectives **COMPETENCY IN MEDICAL KNOWLEDGE:** Inflammation is a key hallmark in ACM pathology. Improved knowledge regarding its role in disease progression can aid early diagnosis and medical management of patients with ACM.

**TRANSLATIONAL OUTLOOK:** Anti-inflammatory therapies such as anti-IL1B antibodies have the potential to expand the limited treatment repertoire for ACM, which currently aims to prevent arrhythmic burden than prevention of myocardial inflammation leading to fibrotic remodeling.

## Funding Support and Author Disclosures

This work was funded by the following: National Institutes of Health grants 5T32AI007163-44 (to Dr Penna), R01-HL148348 (to Dr Saffitz), and R35-HL161185 (to Dr Lavine); American Heart Association Predoctoral Fellowship no. 826325 (to Dr Amrute); American Heart Association Career Development Award no. 19CDA34760185 (to Dr Chelko); Florida State University Institute of Pediatric Rare Diseases (to Dr Chelko); Washington University in St Louis Rheumatic Diseases Research Resource-Based Center grant NIH P30AR073752 (to Dr Lavine); Leducq Foundation Network grant 20CVD02 (to Dr Lavine); Burroughs Wellcome Fund grant 1014782 (to Dr Lavine); Children’s Discovery Institute of Washington University and St Louis Children’s Hospital grants CH-II-2015-462, Ch-II-2017-628, and PM-LI-2019-829 (to Dr Lavine); and Foundation of Barnes-Jewish Hospital grant 8038-88 (to Dr Lavine .) Dr Lavine has served on the Advisory Board for Medtronic; and has received sponsored research agreements from Amgen, Novartis, Implicit Biosciences, and Kiniksa. Dr Saffitz has served as a consultant for Rejuvenate Bio, Implicit Bioscience, and Rocket Pharmaceuticals. Drs Saffitz and Asimaki hold a US Patent (US Patent 10,317,417) for the use of buccal cells in the diagnosis of ACM. Dr Chelko has served on the Advisory Board for Rejuvenate Bio and Who We Play For. All other authors have reported that they have no relationships relevant to the contents of this paper to disclose.
