# Supplementary material for: Interleukin-1β Drives Disease Progression in Arrhythmogenic Cardiomyopathy
Source: JACC Basic Transl Sci. 2026 May 5;11(6):101542. doi: 10.1016/j.jacbts.2026.101542 (PMC13158593; doi:10.1016/j.jacbts.2026.101542)
Supplement: Supplemental Material 1 [file mmc1.docx]

**List of Supplementary Materials**

Supplemental Methods

Figures 1 to 9

Tables 1 to 5

References

Data supplement file

**Supplemental Methods**

*Ethical approval for human samples*

This study is compliant with all relevant ethical regulations and approved by Washington University School of Medicine Institutional Review Board (IRB no. 201104172). Each patient provided informed consent prior to tissue collection, and no compensation was provided for study participation. All patients have been deidentified.

*Human sample inclusion criteria*

Donor myocardial samples were obtained from patients classified with stable ejection fractions, no known history of cardiac disease, and who experienced a non-cardiac related reason for transplant or death (DBD) were provided by Mid America Transplant Service. These hearts had been rejected for use due to blood type incompatibility or age. ACM myocardial samples were derived from patients undergoing heart transplant. Specific ACM pathogenic variants were determined for each sample using the ClinVar database. Only samples that had a single gene variant for ACM were included in this study.

*Ethical approval for Animal Studies*

All experiments conformed to the Guide for the Care and Use of Laboratory Animals from the National Institute of Health (NIH publication no. 85–23, revised 1996). Animal study protocols were approved by the Florida State University (Protocol Code: 202000052; Date of Approval: 02/10/2021) and Washington University in St. Louis (Protocol Code: D1600245, Date of Approval: 12/02/2020) Animal Care and Use Committee. Mice were housed in temperature-controlled rooms (20-22 °C) and humidity (40%-60%) with a 12-hr light/dark cycle and provided *ad libitum* access to standard rodent chow and water. Age-matched C57BL/6 mice served as WT controls. The generation of *Dsg2*^mut/mut^ mice has been previously described.*^1^*

*Human myocardial tissue processing*

Cardioplegia was injected into coronaries, the organ was then put on cold cardioplegia on ice, several 7-10 mm^3^ tissue specimens were harvested from the apical anterior left ventricular wall within 1 hr and flash frozen immediately in liquid nitrogen to be used for downstream RNA sequencing studies. Additional tissue specimens were fixed in 4% paraformaldehyde and then embedded in paraffin for histology and spatial transcriptomics.

*Human 10x single nuclei processing*

Frozen human myocardial samples were prepared for single nuclei RNA sequencing (snRNAseq) as described previously.*^2^* In brief, tissues were minced with a razor blade and transferred to a Dounce homogenizer containing lysis buffer (10x Genomics). Samples were homogenized gently and then incubated on ice for 15 min. Following filtration and washing steps, samples were stained with 1 μL of DRAQ5 (Thermo Scientific Cat No: 62251) and DRAQ5+ nuclei were sorted and counted on a hemocytometer (Thermo Fisher). Nuclei were subsequently processed using the Chromium Single Cell 5’ Reagent V2 kit from 10x Genomics (PN-1000263). A total of 10,000 nuclei per sample were loaded onto the chip for GEM generation. Library preparation was performed according to protocol and sequencing was performed on a NovaSeq 6000 platform (Illumina).

*Human 10x Visium slide preparation*

Human myocardial samples were paraffin-embedded as described above. To determine tissue RNA quality, DV200 values for each sample were determined using the RNeasy FFPE kit (Qiagen Cat No:73504). Samples with a DV200 value greater than 40 were selected for downstream processing. 10-µm sections were placed on spatial gene expression slides (Visium, 10x Genomics, PN-1000187). Samples were processed following the Visium User Guide (CG000239). Brightfield histology images were taken on a Zeiss Axioscan Z1 (Carl Zeiss AG). Libraries were generated according to the Visium user guide and sequenced on a NovaSeq 6000 (Illumina).

*10x single nuclei RNA seq analysis*

Alignment, quality control, and filtering were performed as previously described.*^2^* In brief nuclei were aligned to the human GRCh38 reference using CellRanger v6.1. Subsequent quality control, normalization, dimensional reduction, and clustering were performed in Seurat v4.0. Following normalization, quality control was performed and cells passing the following criteria were kept for downstream processing: 500 < nFeature_RNA < 4,000 and 1,000 < nCount_RNA < 10,000 and percentage mitochondrial reads < 5%. To remove doublets, Scrublet was run with a cutoff score of >0.25 to identify doublets. Following doublet removal, raw RNA counts were normalized and scaled using SCTransform. Principle components (PCs) were then calculated, and an elbow plot was generated to select the cutoff for significant PCs to use for downstream analysis. UMAP dimensional reduction was then computed using the selected significant PCs. Unsupervised clustering was then performed using the FindNeighbors and FindClusters function, again using the selected significant PC level as above, calculating clustering at a range of resolutions between 0.1 and 0.8 at intervals of 0.1. Differential gene expression was performed using the FindAllMarkers command and a Wilcoxon rank-sum test with a log fold change (FC) cutoff of 0.25 and a min.pct cut-off of 0.1. Clusters were annotated using canonical gene and protein markers. To cluster cell types into distinct cell states, the cell type of interest was subsetted, renormalized, computed PCAs, computed UMAPs, and clustered data at a range of resolutions. DE analysis was then used to identify marker genes for each cell state. Using the top marker genes, gene set z-scores were calculated and plotted in UMAP space. Pseudobulk differential gene expression was performed as pervious described.*^3^*

*Visium Spatial Transcriptomics Analysis*

Space ranger was used to align counts, and the following matrices were processed using Seurat v4, stlearn, and tangram. First, tangram was used in conjunction with the snRNA-seq atlas for voxel deconvolution using baseline parameters. Cell type deconvolution scores were overlaid on the spatial histological images to infer spatial localization. Within the Seuratv4 and stlearn pipeline, the data was normalized and clustered with dimensional reduction to identify spatial niches which were characterized using differential gene expression analysis. Spatial correlation analysis was used to identify cell types which co-localize in space—briefly, the tangram deconvolution score matrix was used and a correlation matrix constructed for downstream visualization (for the purposes of the visualization the diagonal was set to 0). For building pie charts in space, we used the tangram deconvolution scores and stlearn to overlap compositional pie charts in space. Progeny was used for pathway analysis across spatial niches.

*Mouse 10x Single Cell preparation*

Samples were prepared as described previously.*^4^* In brief, freshly isolated hearts from 6-week-old WT and *Dsg2*^mut/mut^ mice (n = 3 per group) were minced on ice with a razor blade, transferred to a 15-mL conical tube containing 3 mL DMEM (Gibco Cat No: 11965-084) with 170 µL collagenase IV (Sigma Cat No: C5138-1G) (250U/mL final concentration), 35 µL DNAse1(Sigma Cat No: D4527-40KU) (60 U/mL), and 75 µL hyaluronidase (Sigma Cat No: H3506) (60 U/mL), and incubated at 37 °C for 40 min with gentle agitation. The digestion reaction was then quenched and filtered. Samples were then centrifuged at 4 °C, for 5 min at 1,200 rpm and the supernatant was discarded. Pellets were resuspended in 1 mL ACK Lysis buffer (Gibco, Cat. No. A10492-01) and incubated at room temperature for 5 min, then quenched with DMEM and centrifuged as above. Supernatant was discarded and the pellets were resuspended in 1 mL of FACS buffer and divided evenly by volume before 500 µL of respective antibody staining cocktail (for fibroblasts and myeloid) was added (data supplement). Samples were stained for 30 min at 4° in the dark. Samples were washed with FACS buffer and centrifuged as above and resuspended in 300 µL of FACS buffer. Samples were sorted on a BD FACS Melody and subsequently counted on a hemocytometer. Fibroblasts were identified as PDGRFA+PDPN+CD45-CD31-. Myeloid cells were identified as CD45+CD11b+Ly6G-. Collected cells were processed using the Single Cell 3’ v3.1 kit (10x Genomics PN: 1000268). 10,000 cells from each sample were loaded onto the chip for GEM generation. Library preparation was performed according to protocol and sequencing was performed on a NovaSeq 6000 platform (Illumina).

*10x single cell RNA seq analysis*

Alignment, quality control, and filtering were performed as previously described.*^3,4^* Cells were aligned to the mouse GRCh38 using CellRanger v6.1. Subsequent filtering and quality control was performed as above using the following filters for fibroblasts and myeloid cells respectively: 500 < nFeature_RNA < 6,000 and 1,000 < nCount_RNA < 25,000 and percentage mitochondrial reads < 5%, 500 < nFeature_RNA < 6,000 and 1,000 < nCount_RNA < 25,000 and percentage mitochondrial reads < 10%. Normalization and clustering were performed as above.

*Pathway analysis*

Pathway analysis was performed as previously described using EnrichR (<http://maayanlab.cloud/Enrichr/>).*^3,4^*

*Immunofluorescence staining*

Human and mouse myocardial samples were fixed overnight in 4% paraformaldehyde and then embedded in paraffin blocks. 10-µm sections were cut and placed onto charged slides. For immunofluorescence staining we followed the Opal IHC assay guide (Akoya Biosciences) and used the following primary antibodies: anti-CD68 (BioRad, clone KP1), anti-CCR2 (Abam, clone 7A7), anti-pCASP1 at serine 376 (Fisher Scientific, Cat. No. PA5-38564), anti-NLRP3 (Abcam, Cat. No. ab214185), anti-IL-1β (R&D Systems, Cat. No. AF-401-NA), anti-POSTN (Abcam, Cat. No. ab215199), and anti-fibroblast activation protein (FAP, Abcam clone EPR20021). Images were captured on a Zeiss Axioscan 7 and analyzed in Zen Blue. A full list of antibodies used, their dilutions, and the experimental applications employed (immunofluorescence, immunoperoxidase, and/or flow cytometry) can be found in the Supplement.

*Immunoperoxidase staining*

Formalin-fixed, paraffin-embedded mouse hearts were analyzed by immunoperoxidase staining using a primary antibody against RelA/p65. Sections (5-μm thick) were deparaffinized, dehydrated, rehydrated, and exposed to 3% hydrogen peroxide solution for 10 min to block endogenous peroxidase activity. Sections were first incubated with 5% donkey serum (Stratech Scientific, Cat. No. 017-000-121) and 5% BSA (Merck Life Science, Cat. No. A2153) in 1X TBS blocking solution for 1 hr then overnight at 4 °C with rabbit anti-RelA polyclonal antibody (LSBiosciences Cat. No. LS-B653; at 1:100). The following day, sections were incubated with horseradish peroxidase donkey anti-rabbit secondary antibody (Thermo Fisher Scientific, Cat. No. A16038, at 1:400) for 1 hr at room temperature. Peroxidase-conjugated antibodies were detected by the 3,3'-diaminobenzidine (DAB) substrate kit (Abcam, Cat. No. ab64238). Slides were then counterstained with Mayer’s hematoxylin (CELLAVISION, Cat. No. 361075) and bright field images were taken with a Nikon Eclipse 80i microscope and recorded with Nikon DS-Fi1 camera. The number of cardiac myocytes showing nuclear signal for RelA were counted in 5 regions of interest (ROIs) and expressed as the number of cells/mm^2^.

*Single-labeling immunofluorescence staining:*

Mouse myocardial sections were analyzed by immunofluorescence staining using primary antibodies against junctional plakoglobin (JUP) and connexin-43 (Cx43). Sections (5-μm thick) were deparaffinized, dehydrated, rehydrated, and boiled in citrate buffer (pH 6) for 10 min. Slides were incubated with 3% goat blocking solution (Stratech Scientific, Cat. No. 005-000-121), containing 1% BSA (Merck Life Science, Cat. No. A2153) and 0.15% Triton-X (Thermo Fisher Cat. No. A16046.0F) in 1X PBS for 1 hr and then rabbit anti-JUP monoclonal antibody (Abcam, Cat. No. Ab184919, at 1:100) or rabbit anti-Cx43 polyclonal antibody (Sigma Aldrich, Cat. No. C6219, at 1:200) overnight at 4 °C. The following day, sections were incubated with anti-rabbit Cy3-labeled secondary antibody (Jackson Immunoresearch Cat. No. 111-165-144 at 1:400) and then mounted with ProLong Gold. Images were obtained using a Nikon A1R confocal microscope.

*Double-labeling immunofluorescence staining:*

Mouse myocardial sections were analyzed by double-labeling immunofluorescence staining using primary antibodies against CCR2 and CD68. Briefly, sections (5-μm thick) were incubated at 60° (20 min), deparaffinized, dehydrated, rehydrated and boiled in citrate buffer (pH 6) for 11 min. Slides were then washed in 1X PBS (3x, 5 min/wash), incubated with 5% donkey serum (Stratech Scientific, Cat. No. 017-000-121), 1% BSA (Merck Life Science, Cat. No. A2153) and 0.15% Triton-X (Thermo Fisher, Cat. No. A16046.0F) in 1X PBS for 1 hr and then probed with rat anti-CD68 antibody (Thermo Fisher, Cat. No. 14-0681-82, at 1:200) and rabbit anti-CCR2 antibody (Thermo Fisher, Cat. No. BS-23026R, at 1:400) overnight at 4°. The following day, sections were incubated with anti-rat FITC-conjugated and anti-rabbit Cy5-conjugated secondary antibodies (Invitrogen Cat. No. A31573 at 1:500 and Cat. No. A21208 at 1:500 respectively) and mounted with ProLong Gold (Thermo Fisher, Cat. No. P10144). Images were obtained using a Nikon A1R confocal microscope. The number of macrophages showing strong immunofluorescent signal for CCR2 were counted in 5 ROIs and expressed as the number of cells/mm^2^.

*Animal Echocardiography*

Cardiac function was assessed prior to and at treatment endpoint using the Vevo F2 Imaging Platform (Fujifilm Visualsonics, Washington). The Vevo F2 imager was utilized to obtain both short- and long-axis images at the level of the papillary muscles (sweep speed of 200 mm/s), as previously described.*^1,5^* Images were analyzed using the American Society of Echocardiography guidelines for animals.*^6^* Three to 5 images were obtained for each mouse/timepoint, then averaged to assess %LVEF and wall/chamber dimensions.

*Animal ECGs*

Mice were anesthetized via nose cone anesthesia (1.5%-2% isoflurane vaporized in 100% O_2_) and ECG electrodes were placed between the right and left front paws while the mouse was in a supine position to obtain Lead I ECG recordings, as previously described.*^5^* iWorx 8 lead channel (iWorx Bio-8, New Hampshire) with ECG Analysis Add-on Software was utilized to analyze Signal-averaged ECGs (SAECGs) from 10-min recordings. ECG Analysis Add-on Software afforded us to measure the following parameters: wave durations, intervals, and wave amplitudes (via SAECGs). Percent premature ventricular contractions (%PVCs) were analyzed via totaling the number PVCs throughout the 10-min recording then divided by the number of total beats, times 100. Following terminal ECG recordings (ie, at 16 or 24 weeks of age), mice were euthanized and hearts excised for downstream pathological, protein/mRNA, and sequencing analyses.

*Masson’s Trichrome Staining*

Hearts were formalin-fixed, paraffin-embedded (FFPE) and blocks cut at 5 µm (2-3 cuts per slide) and stained with either Masson’s Trichrome (Sigma; Cat. No. HT15-1KT) or Hematoxylin and Eosin (H&E Kit; Abcam, Cat. No. ab245880) following the manufacturer’s protocol. Myocardial sections were traced using ImageJ to achieve total area. Then blue fibrotic sections were traced to record total fibrosis. Percent myocardial fibrosis was determined by the sum of all fibrotic areas (within one slice) divided by total myocardial area using ImageJ version 1.53e software. Each slice was then averaged to obtain percent myocardial fibrosis for one mouse.

*Mouse single nuclei preparation for iCellcx8 sequencing*

Nuclei were isolated from frozen mouse heart samples from the early intervention anti-IL-1β treatment experiment using the Chromium Nuclei Isolation kit from 10x Genomics (PN: 100047), stained with DRAQ5. Following staining, DRAQ5+ nuclei were sorted and counted on a hemocytometer. Subsequently we utilized and followed the SMART-Seq Pro Application kit (Takara Biosciences) to prepare samples for dispense by the iCell8cx system and subsequent reverse transcription, cDNA amplification, and library generation. Libraries were sequenced on one full lane of NovaSeq X Plus.

*iCellcx8 library alignment*

iCellcx8 sequencing data was aligned to the mouse reference genome via Cogent AP (Takara Biosciences). Following alignment gene matrix files were generated for each library. Gene matrices were converted into an acceptable format for Seuratv4 using R software (R Core Team (2025). *R: A Language and Environment for Statistical Computing*. R Foundation for Statistical Computing, Vienna, Austria. https://www.R-project.org/). Subsequent analysis was performed as above for single nuclei samples.

**
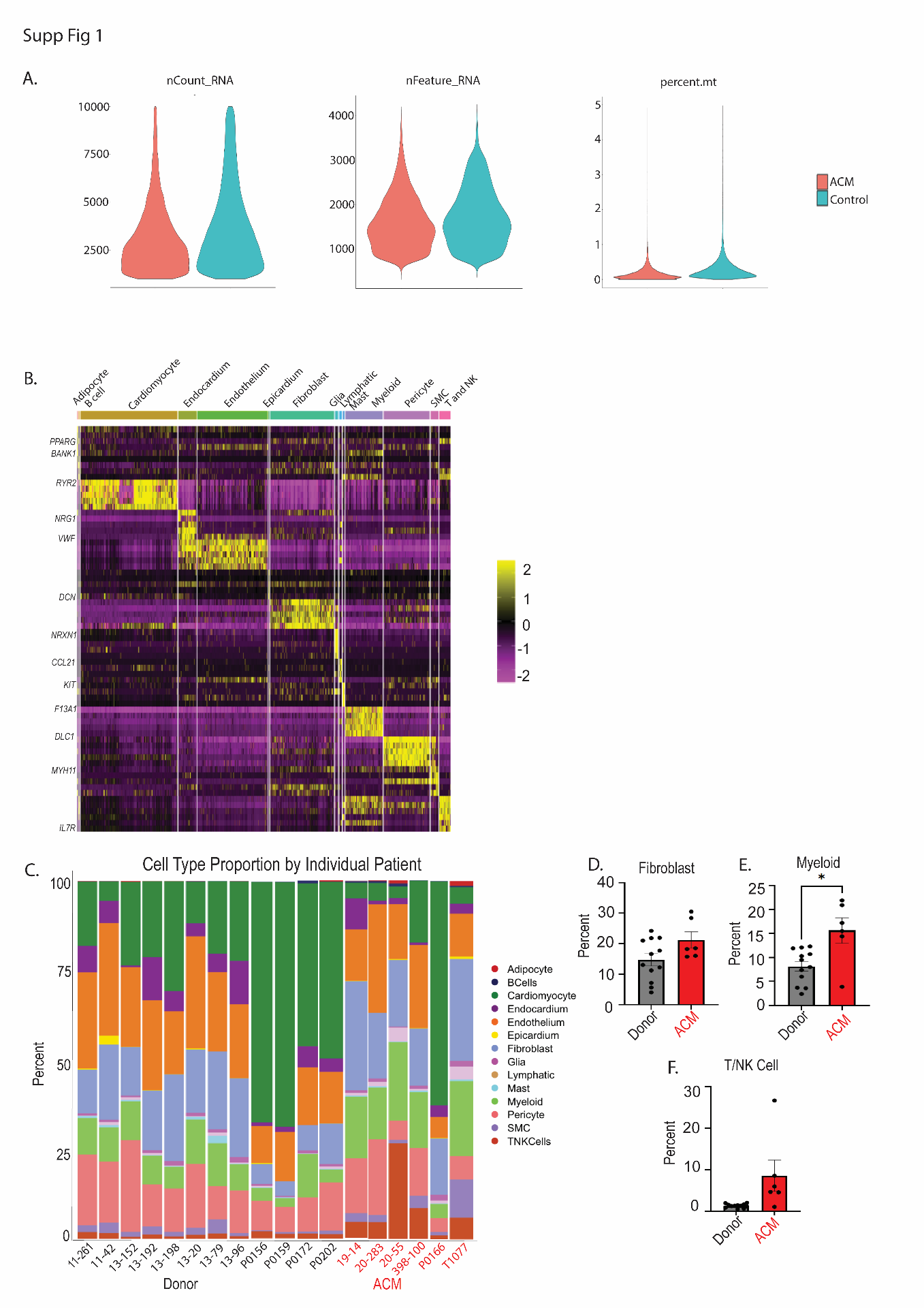
**

**Supplemental Figure 1. QC metrics for human myocardial samples.** (**A**) Major QC cutoffs for single nuclei RNA sequencing data. RNA count: 1,000 < n < 10,000, Feature count: 500 < n < 4,000, Mitochondrial percentage: n < 5. (**B**) Heatmap displaying major canonical gene expression across different cell types. (**C**) Composition plot displaying proportion of major cell types across individual samples. (**D**) Quantification of fibroblast proportion comparing donor samples to ACM samples (n = 12 and n = 6, respectively). (**E**) Quantification of myeloid proportions comparing donor samples to ACM samples (n = 12 and n = 6, respectively). (**F**) Quantification of T/NK cell proportion comparing donor samples to ACM samples (n = 12 and n = 6, respectively). Brown-Forsythe and Welch ANOVA testing was used for graphs from (**D**), (**E**), and (**F**). Data are presented as mean ± SEM. **P* < 0.05, ***P* < 0.01 and ****P* < 0.001.


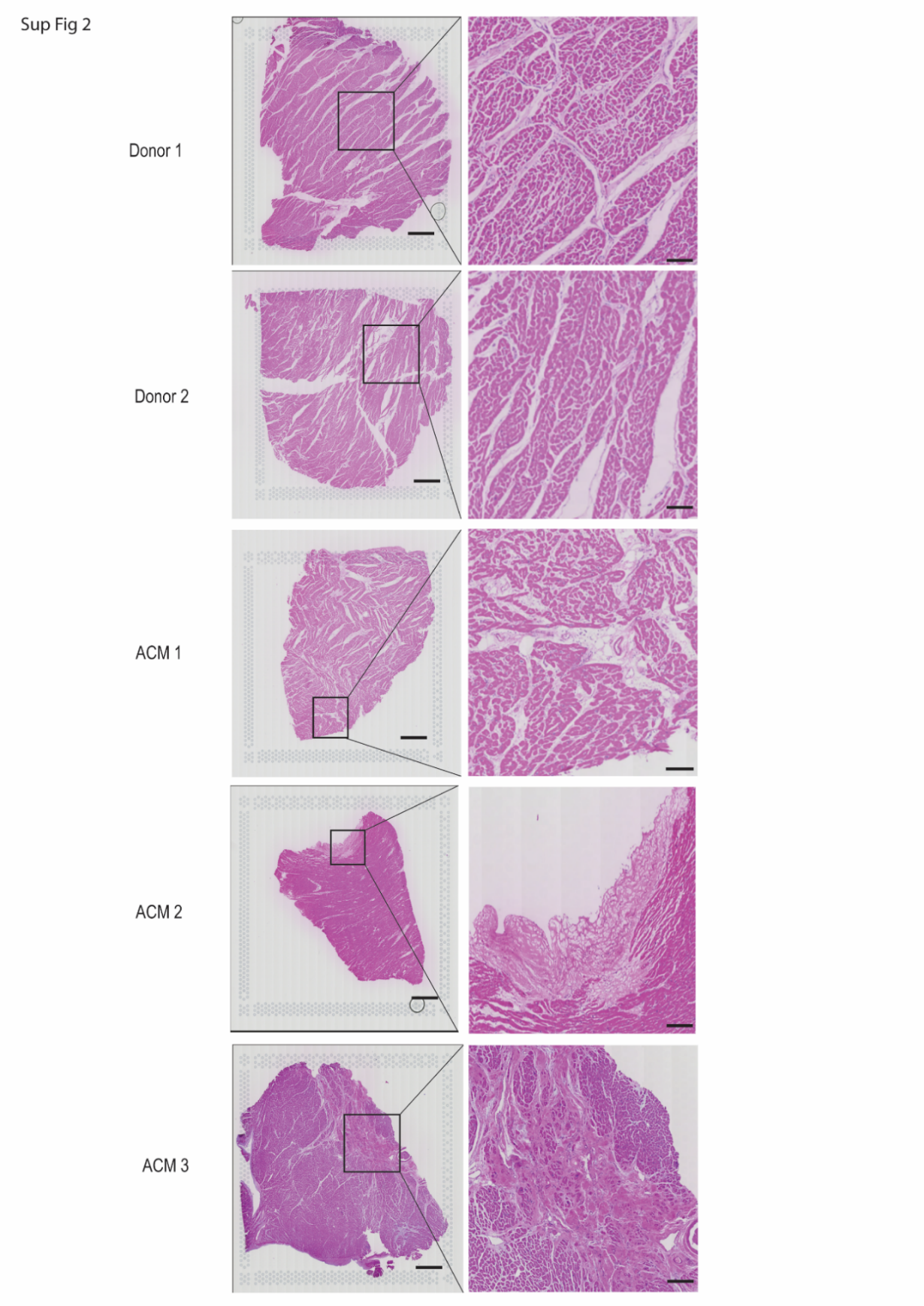


**Supplemental Figure 2. H&E images used for Visium spatial transcriptomics.** H&E images for each sample that were used for spatial transcriptomics. Left panel: H&E immunostained myocardium from donor controls and patients with ACM were captured on an Axioscan Z7; scale bar, 200 μm. Right panel: zoomed insets of areas of healthy myocardium (in donor samples) or areas of ACM lesions (in ACM samples); scale bar, 750 μm.


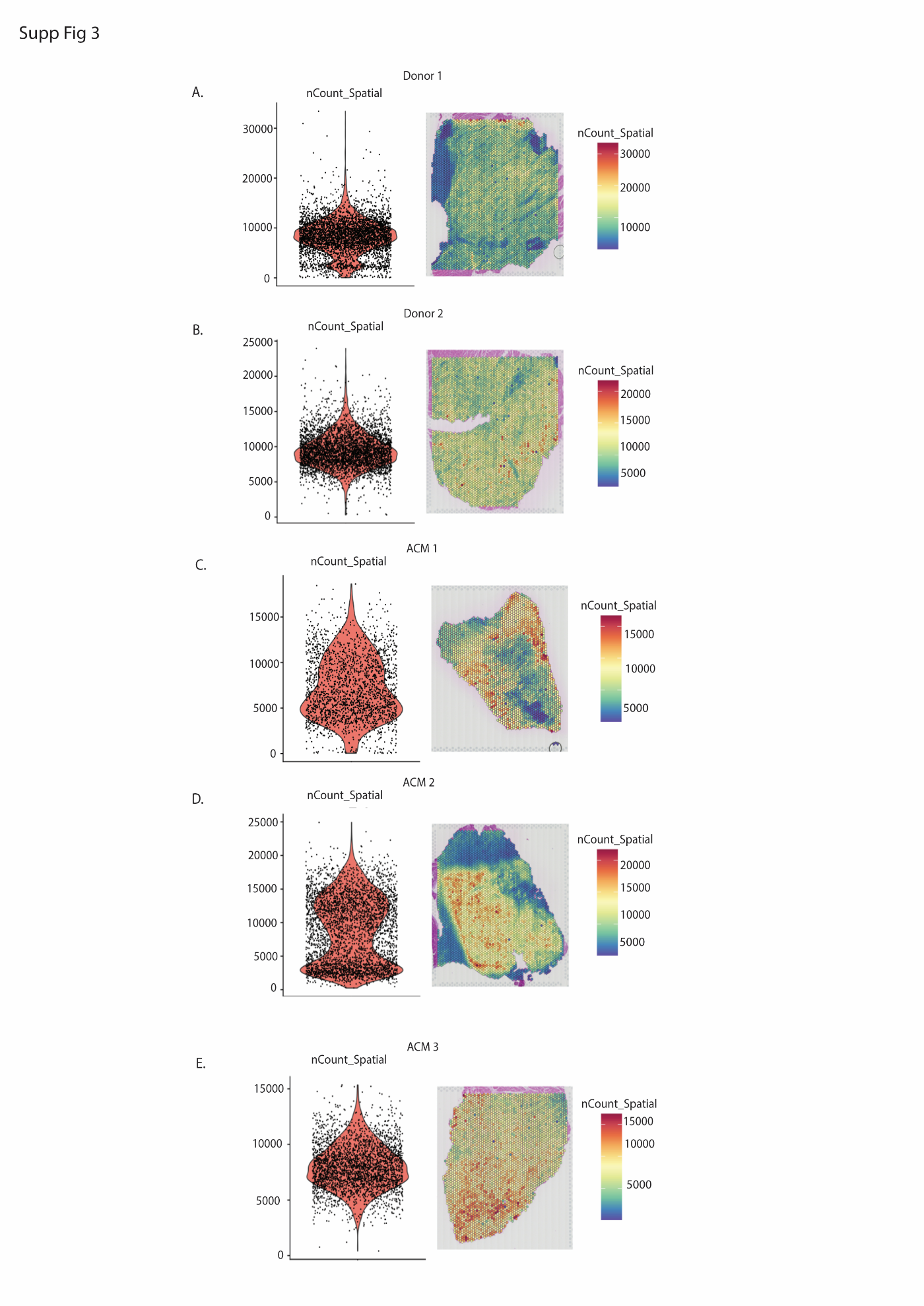


**Supplemental Figure 3. QC metrics for spatial transcriptomics samples.** Spatial UMI counts for each sample: (**A**) Donor 1, (**B**) Donor 2, (**C**) ACM 1, (**D**) ACM 2, (**E**) ACM 3.


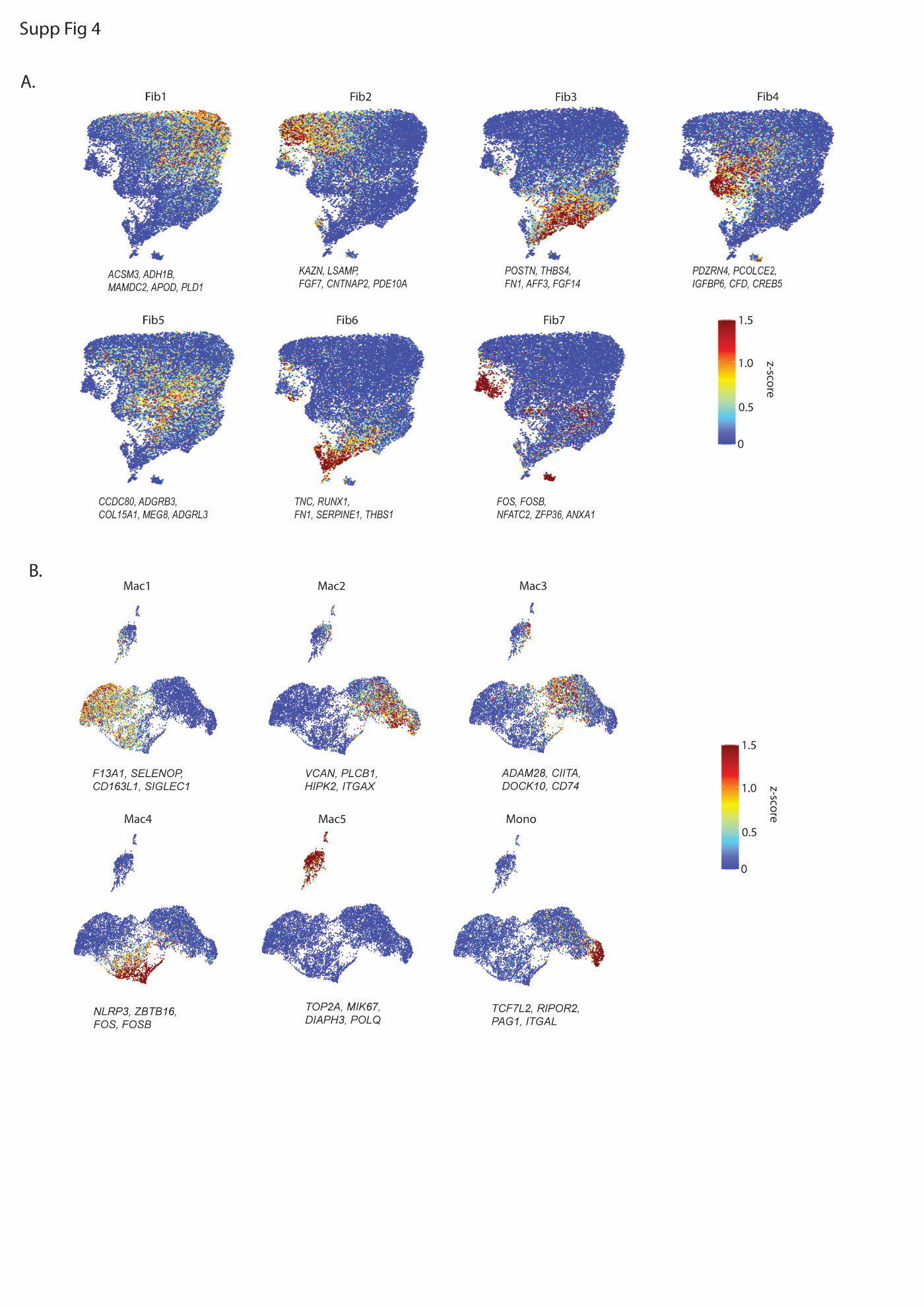


**Supplemental Figure 4. Sub population z-scores for human fibroblasts and myeloid cells.** (**A**) Fibroblast sub populations identified by major gene expression. (**B**) Myeloid sub populations identified by major gene expression.


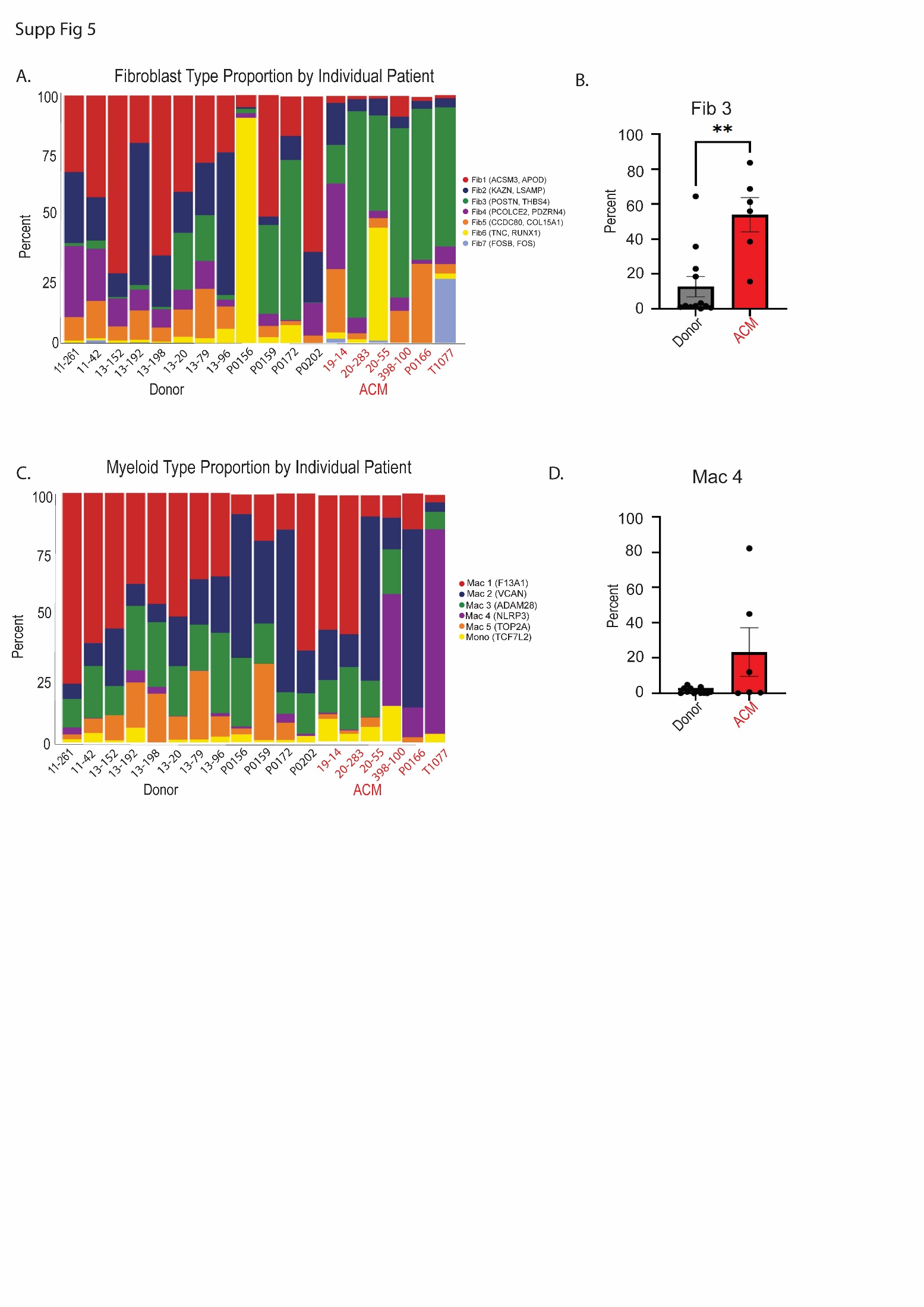


**Supplemental Figure 5. Proportions of fibroblast and myeloid subtypes by individual sample.** (**A**) Composition plot displaying proportion of major fibroblast cell states across individual samples. (**B**) Quantification of the Fib3 proportion between donor and ACM samples (n = 12 and n = 6, respectively). (**C**) Composition plot displaying proportion of major myeloid cell states across individual samples. (**D)** Quantification of the Mac4 proportion between donor and ACM samples (n = 12 and n = 6, respectively). Welch’s *t*-test was used for graphs from (**B**) and (**D**). Data are presented as mean ± SEM. **P* < 0.05, ***P* < 0.01, and ****P* < 0.001.


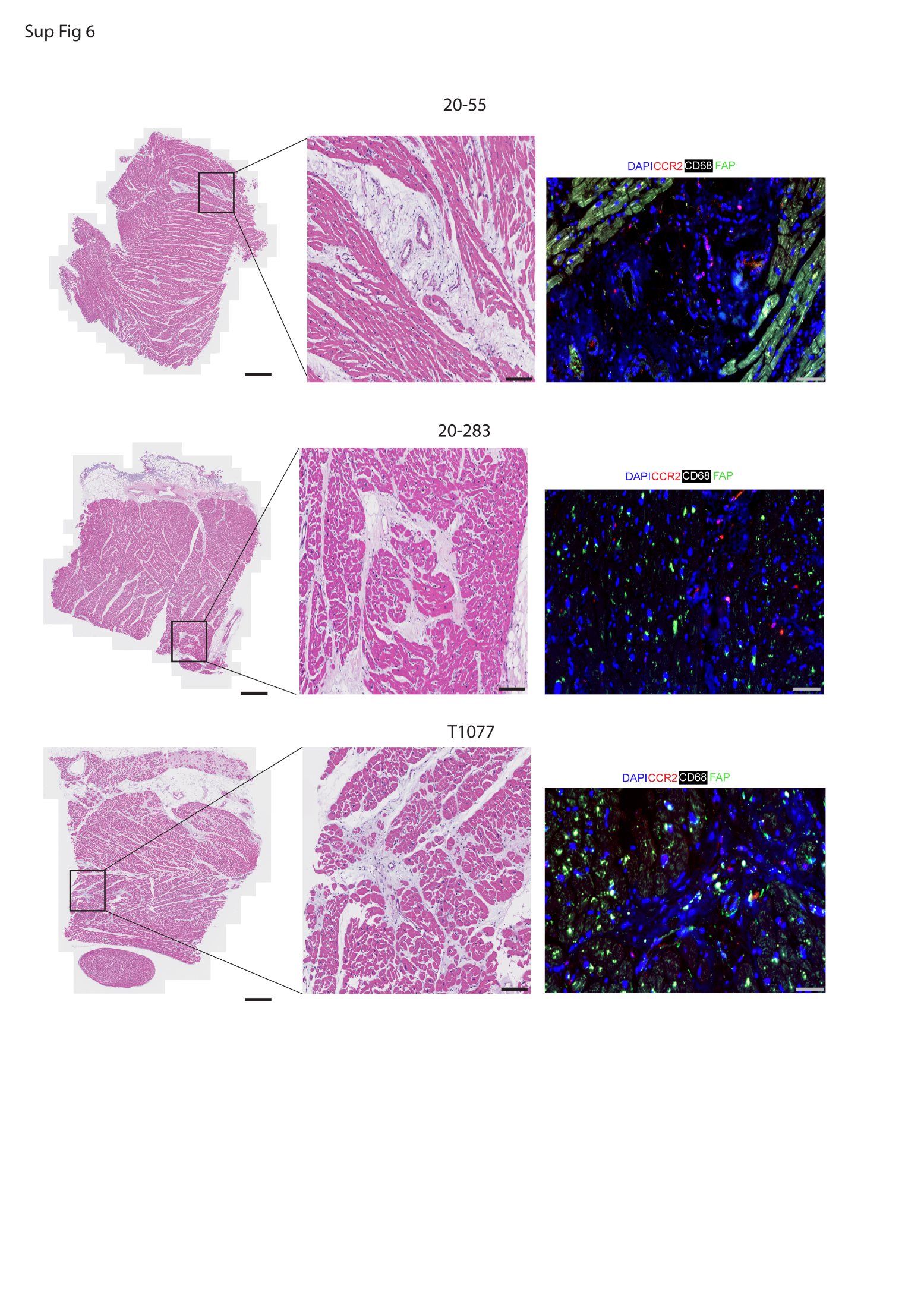


**Supplemental Figure 6. H&E and immunofluorescence staining in RV samples reveal similar colocalization of inflammatory macrophages and activated fibroblasts in ACM lesions.** Left panel: H&E immunostained myocardium from the right ventricle of a subset of human samples used for our overall analysis; scale bar, 200 μm. Middle panel: magnified insets of areas of lesions; scale bar, 750 μm. Right panel: Immunofluorescence staining displaying colocalization of CCR2+ and CD68+ macrophages and FAP+ fibroblasts in these areas of lesions; scale bar, 50 μm.


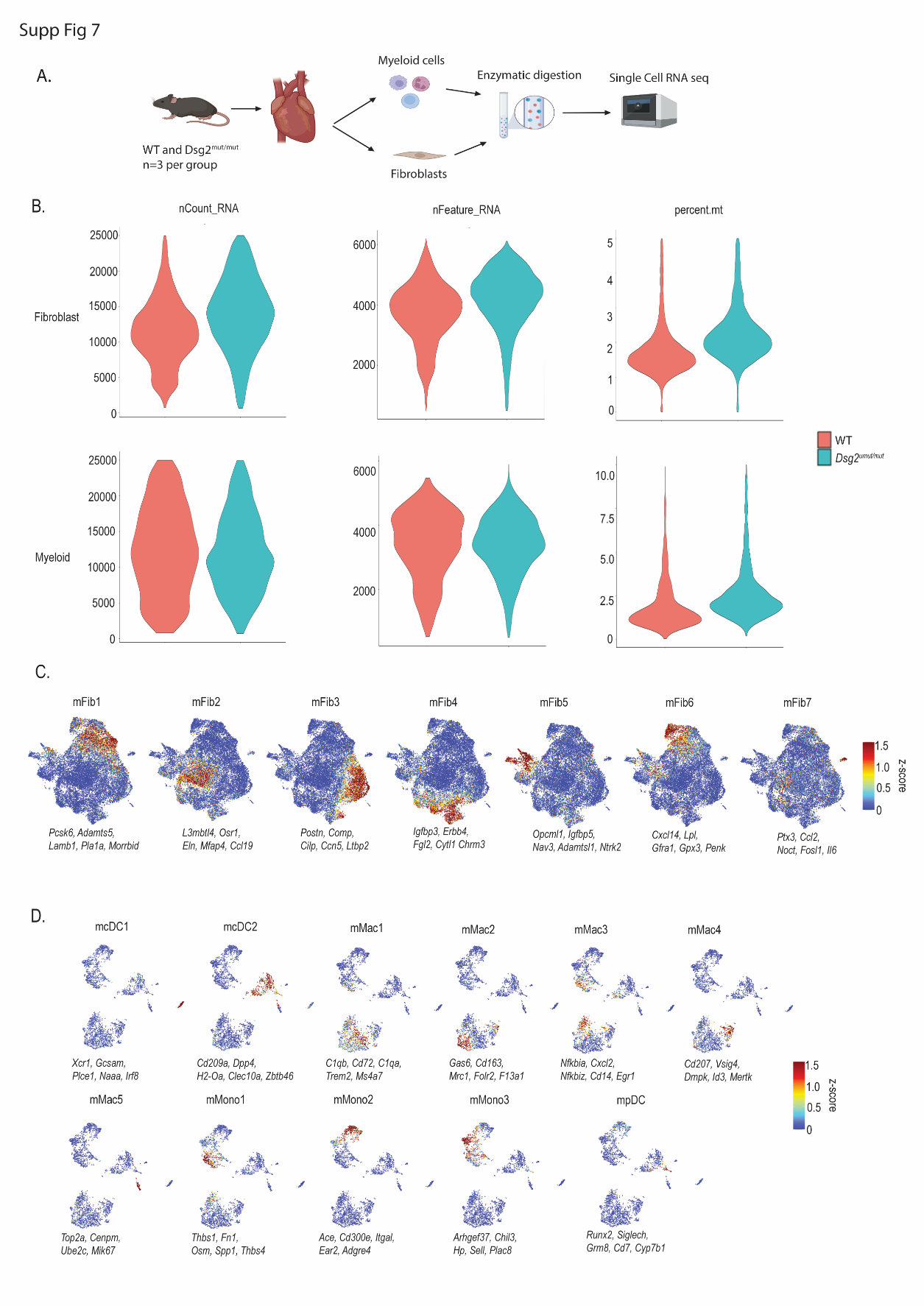


**Supplemental Figure 7. Mouse single cell RNA sequencing QC metrics and major population z-scores.** (**A**) Study design outlining how myeloid and fibroblast libraries were sequenced. (**B**) Major QC cutoffs for single cell RNA sequencing data. RNA count: 1,000 < n < 25,000, Feature count: 500 < n < 6,000, Mitochondrial percentage: n < 5 for fibroblasts and n < 10 for myeloid. (**C**) Mouse fibroblast sub populations identified by major gene expression. (**D**) Mouse myeloid sub populations identified by major gene expression.


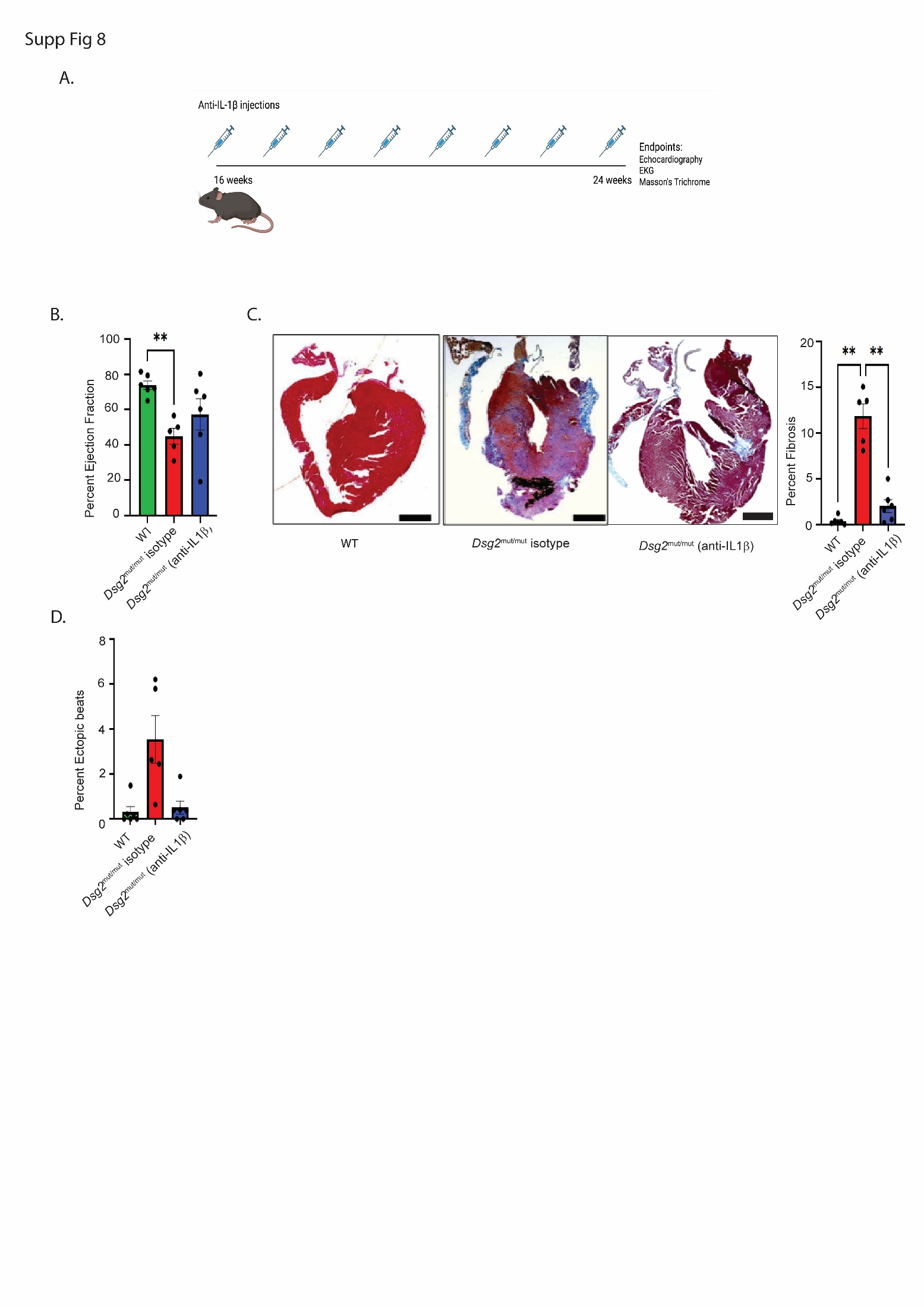


**Supplemental Figure 8. Late IL-1β blockade in *Dsg2*^mut/mut^ mice.** (**A**) Study design outlining treatment schedule of neutralizing IL-1β antibody. (**B**) Measurement of ejection fraction (n = 6, n = 5, and n = 6 for each group, respectively). (**C**) Representative Masson’s trichrome images from each treatment group and quantification of fibrosis percentage (n = 6, n = 5, and n = 6 for each group, respectively); scale bar, 1 mm. (**D**) Quantification of percentage of ectopic beats (n = 6, n = 5, and n = 6 for each group, respectively). Brown-Forsythe and Welch’s ANOVA testing was used for graphs from (**B**), (**C**) and (**D**). Data are presented as mean ± SEM. **P* < 0.05, ***P* < 0.01, and ****P* < 0.001.


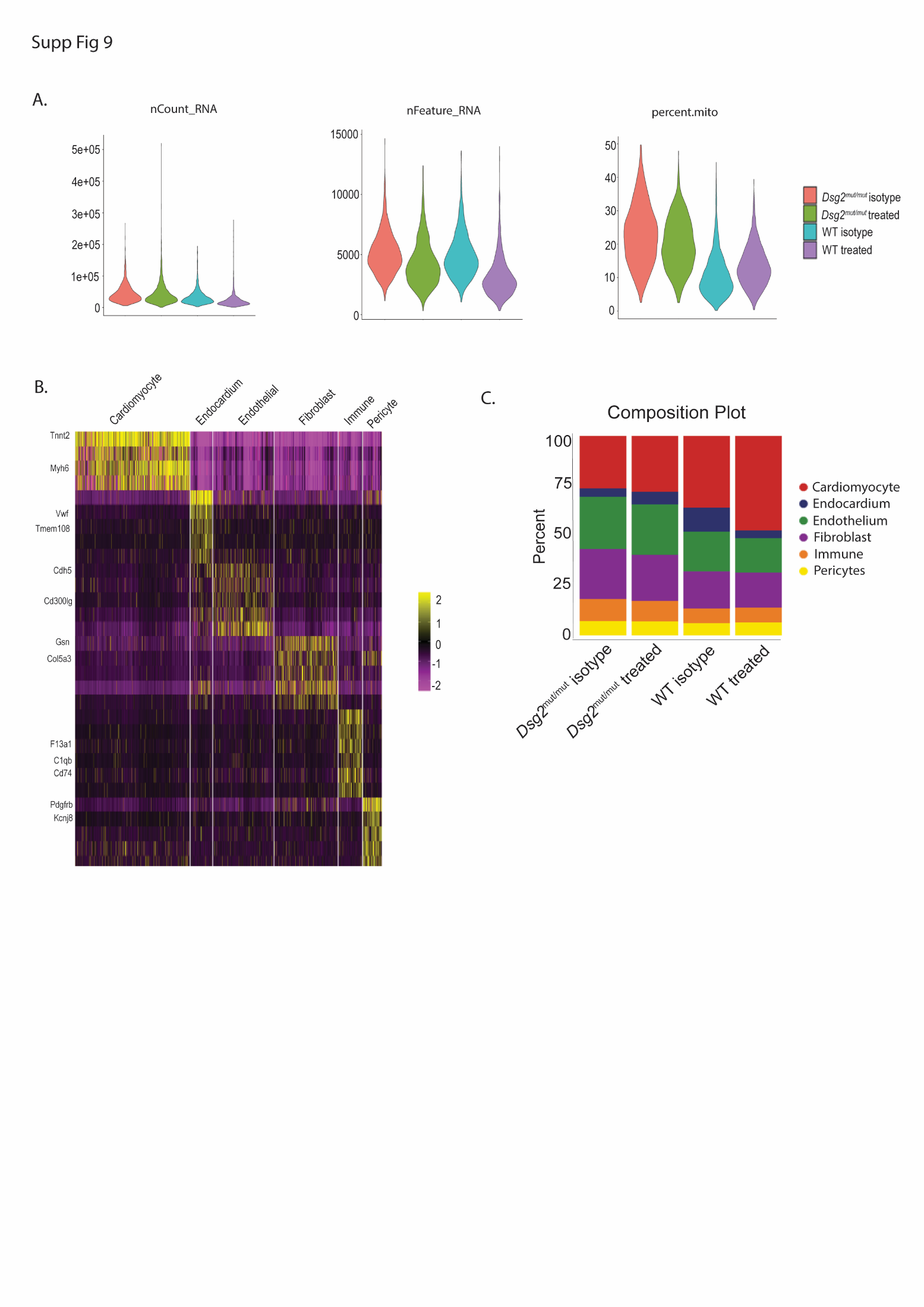


**Supplemental Figure 9. Mouse single nuclei RNA sequencing with iCell8cx system.** (**A**) Major QC cutoffs for single cell RNA sequencing data. RNA count: 1,000 < n < 300,000, Feature count: 500 < n < 20,000, Mitochondrial percentage: n < 30. (**B**) Heatmap displaying major canonical gene expression across different cell types. (**C**) Composition plots comparing major cell populations across treatment groups.

**Supplemental Table 1. List of human samples used for single nuclei sequencing and spatial transcriptomics.**

| **Sample** |  | **Condition/Mutation** | **Gender** | **Age (years)** | **DV200 (%)** | **Used for spatial** | **Mutation** |
| --- | --- | --- | --- | --- | --- | --- | --- |
| 11-42 |  | Donor control | F | 48 | 39 |  | N/A |
| 11-261 |  | Donor control | F | 37 | 43 | X | N/A |
| 13-20 |  | Donor control | M | 45 | 37 |  | N/A |
| 13-192 |  | Donor control | F | 21 | 35 |  | N/A |
| 13-198 |  | Donor control | M | 11 | 38 |  | N/A |
| 13-79 |  | Donor control | F | 19 | 42 | X | N/A |
| 13-96 |  | Donor control | M | 28 | 39 |  | N/A |
| 13-152 |  | Donor control | M | 35 | 30 |  | N/A |
| P0202 |  | Donor control | F | 8 | 32 |  | N/A |
| P0172 |  | Donor control | M | 7 | 34 |  | N/A |
| P0156 |  | Donor control | F | .75 | 36 |  | N/A |
| P0159 |  | Donor control | M | 1 | 33 |  | N/A |
| 19-14 |  | *PKP2* | M | 46 | 46 | X | Stop gain  Exon 6  1475_1476insTT  p.R493* |
| 20-55 |  | *DSP* | F | 13 | 38 |  | Missense  Exon 17  c.G2423A  p.R808H |
| 20-283 |  | *DSP* | M | 14 | 44 | X | Stop gain Exon 4  c.C478T  p.R160* |
| P0166 |  | *PKP2* | NA | NA | 35 |  | Stop gain  Exon 8  c.C1819T  p.R607* |
| 398-100 |  | *PKP2* | F | NA | 54 | X | 2013delC |
| T1077 |  | *DSP* | M | 61 | 32 |  | Frameshift  Exon 24  c.3882_3885del  p.S1295Lfs*34 |

*PKP2*, Plakophilin-2; *DSP*, Desmoplakin.

**Supplemental Table 2. Clinical characteristics from patients with ACM.**

| **Sample** | **Structural & Morpho-functional Abnormalities** | **Repolarization abnormalities** | **Depolarization abnormalities** | **Arrhythmias** | **Family History** |
| --- | --- | --- | --- | --- | --- |
| 19-14 | (M)RV; (m)LV | (m) | NN | (M) | NN |
| 20-55 | (m)RV; (m)LV | NN | NN | NN | NN |
| 20-283 | (m)LV | (m) | NN | NN | NN |
| P0166 | NN | NN | NN | NN | NN |
| 398-100 | (M)RVFW | (M) | (M) | (M) | (M) |
| T1077 | NN | NN | NN | NN | NN |

NN, none noted either in pathology and/or clinical report; (M) or (m), meets major or minor Task Force Criteria, respectively; RV/LV, right/left ventricle respectively; RVFW, RV free wall.

**Supplemental Table 3. List of human fibroblast genes (and mouse orthologs) upregulated in ACM used to build gene signature score.**

| **Human Gene Symbol** | **Mouse ortholog** |
| --- | --- |
| *AFF3* | *Aff3* |
| *SMOC2* | *Smoc2* |
| *TENM4* | *Tenm4* |
| *PDE5A* | *Pde5a* |
| *PLCB4* | *Plcb4* |
| *IL1RAPL1* | *Il1rapl1* |
| *SGIP1* | *Sgip1* |
| *PALLD* | *Palld* |
| *IGF1* | *Igf1* |
| *FAP* | *Fap* |
| *DDAH1* | *Ddah1* |
| *ASPN* | *Aspn* |
| *RUNX2* | *Runx2* |
| *MXRA5* | *Mxra5* |
| *LTBP2* | *Ltbp2* |
| *THBS4* | *Thbs4* |
| *MEOX1* | *Meox1* |
| *FN1* | *Fn1* |
| *POSTN* | *Postn* |
| *RUNX1* | *Runx1* |
| *CILP* | *Cilp* |
| *MEOX2* | *Meox2* |
| *NREP* | *Nrep* |
| *CCN2* | *Ccn2* |

**Supplemental Table 4. List of human myeloid genes (and mouse orthologs) upregulated in ACM used to build gene signature score.**

| **Human Gene Symbol** | **Mouse ortholog** |
| --- | --- |
| *WASHC2A* | *Washc2* |
| *NLRP3* | *Nlrp3* |
| *ZBTB16* | *Zbtb16* |
| *FOS* | *Fos* |
| *CSGALNACT1* | *Csgalnact1* |
| *SH3BP5* | *Sh3bp5* |
| *LMNA* | *Lmna* |
| *EMP1* | *Emp1* |
| *GLIS3* | *Glis3* |
| *ANXA1* | *Anxa1* |
| *ELL2* | *Ell2* |
| *FOSB* | *Fosb* |
| *ACSL1* | *Ascl1* |
| *SRGAP1* | *Srgap1* |
| *ARHGEF10L* | *Arhgef10l* |
| *NAMPT* | *Nampt* |
| *PAPSS2* | *Papss2* |
| *TFRC* | *Tfrc* |
| *SLC1A3* | *Slc1a3* |
| *MAN1A1* | *Man1a* |
| *CPM* | *Cpm* |
| *SRGN* | *Srgn* |
| *IGSF21* | *Igsf21* |

**Supplemental Table 5. Selected list of pro-inflammatory and pro-fibrotic cytokines changed following early IL1β blockade in *Dsg2*^mut/mut^ mice.**

|  | **Cytokine** | ***Dsg2*^mut/mut^ (Isotype)** vs **WT (Isotype)** | | ***Dsg2*^mut/mut^ (1mg/kg IL-1β)** vs **WT (Isotype)** | |
| --- | --- | --- | --- | --- | --- |
|  |  |  |  |  |  |
|  |  | **Fold Change** | **P-Values** | **Fold Change** | **P-Values** |
|  | CCL6 | **4-8** | **P<0.001** | **1-2** | **0.87** |
|  | CCL22 | **2-4** | **P<0.050** | **≤1** | **0.99** |
| **Fold Change** | CD14 | **4-8** | **P<0.050** | **1-2** | **0.63** |
|  | CD40 | **8-12** | **0.11** | **2-4** | **P<0.050** |
|  | CXCL2 | **8-12** | **P<0.050** | **4-8** | **0.098** |
| **≤1** | CXCL9 | **2-4** | **P<0.050** | **1-2** | **0.57** |
| **1-2** | IFNγ | **8-12** | **P<0.010** | **2-4** | **P<0.050** |
| **2-4** | IGFBP-2 | **4-8** | **P<0.050** | **1-2** | **0.14** |
| **4-8** | IGFBP-3 | **2-4** | **P<0.050** | **≤1** | **0.95** |
| **8-12** | IL-1β | **8-12** | **P<0.010** | **2-4** | **0.35** |
| **Value** | IL-3 | **≤1** | **0.94** | **2-4** | **0.25** |
|  | MPO | **4-8** | **P<0.050** | **≤1** | **0.30** |
|  | OPN | **19** | **P<0.010** | **2-4** | **0.45** |
|  | POSTN | **4-8** | **P<0.050** | **2-4** | **0.43** |

A full list of cytokines and chemokines assessed (>100) is available in data supplement. Welch’s *t*-test was used for comparisons between cohorts.

**References**

1. Chelko SP, Asimaki A, Andersen P, et al. Central role for GSK3β in the pathogenesis of arrhythmogenic cardiomyopathy. *JCI Insight*. 2016;1.

2. Koenig AL, Shchukina I, Amrute J, et al. Single-cell transcriptomics reveals cell-type-specific diversification in human heart failure. *Nature Cardiovascular Research*. 2022;1:263-280.

3. Amrute JM, Luo X, Penna V, et al. Targeting the Immune-Fibrosis Axis in Myocardial Infarction and Heart Failure. *bioRxiv*. 2022:2022.10.17.512579.

4. Chelko SP, Penna VR, Engel M, et al. NFĸB signaling drives myocardial injury via CCR2+ macrophages in a preclinical model of arrhythmogenic cardiomyopathy. *Journal of Clinical Investigation*. 2024;134.

5. Chelko SP, Asimaki A, Lowenthal J, et al. Therapeutic Modulation of the Immune Response in Arrhythmogenic Cardiomyopathy. *Circulation*. 2019;140:1491-1505.

6. Syed F, Diwan A, Hahn HS. Murine Echocardiography: A Practical Approach for Phenotyping Genetically Manipulated and Surgically Modeled Mice. *Journal of the American Society of Echocardiography*. 2005;18:982-990.
