# Supplementary material for: Interleukin-1β Drives Disease Progression in Arrhythmogenic Cardiomyopathy
Source: JACC Basic Transl Sci. 2026 May 5;11(6):101542. doi: 10.1016/j.jacbts.2026.101542 (PMC13158593; doi:10.1016/j.jacbts.2026.101542)
Supplement: Supplemental Material 2 [file mmc2.pdf]

Uncropped, Unedited Mouse XL Cytokine Arrays

(R&D Systems, Catalog Number ARY028)

Mouse XL Cytokine Array Coordinates

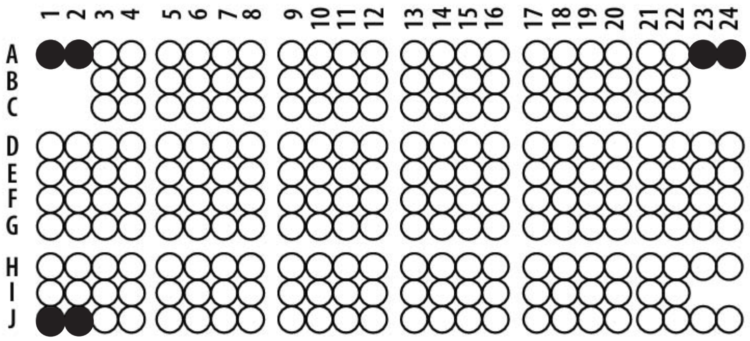

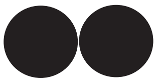

Reference Bands

All cytokines/chemokines are measured in duplicate. The specific targets that correspond with the grid on the left can be found here:

https://www.rndsystems.com/products/proteome-profiler-mouse-xl-cytokine-array_ary028#technical-data

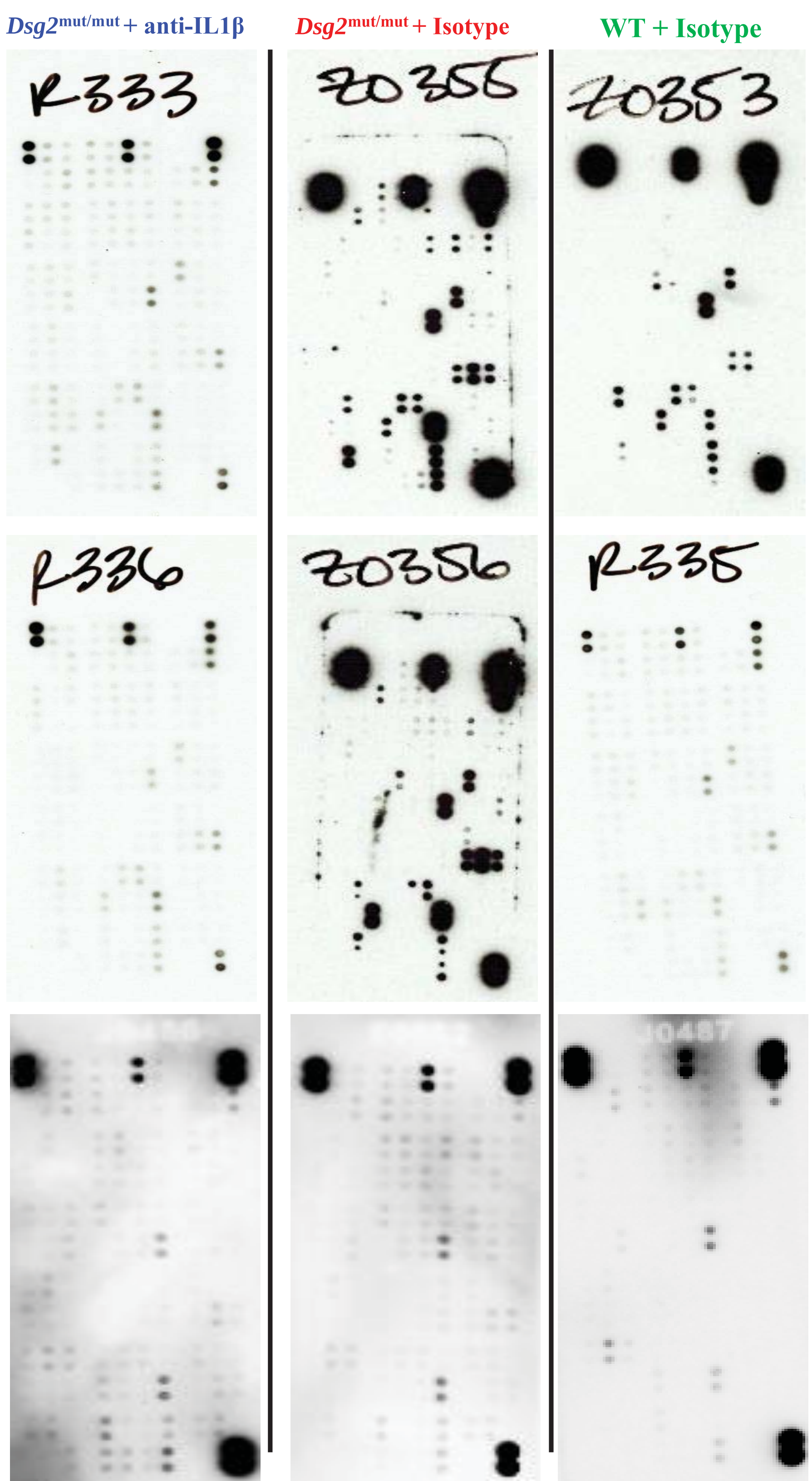

Dsg2mut/mut + anti-IL1β
Dsg2mut/mut + Isotype
WT + Isotype
R333
Z0355
Z0353
R336
Z0356
R335

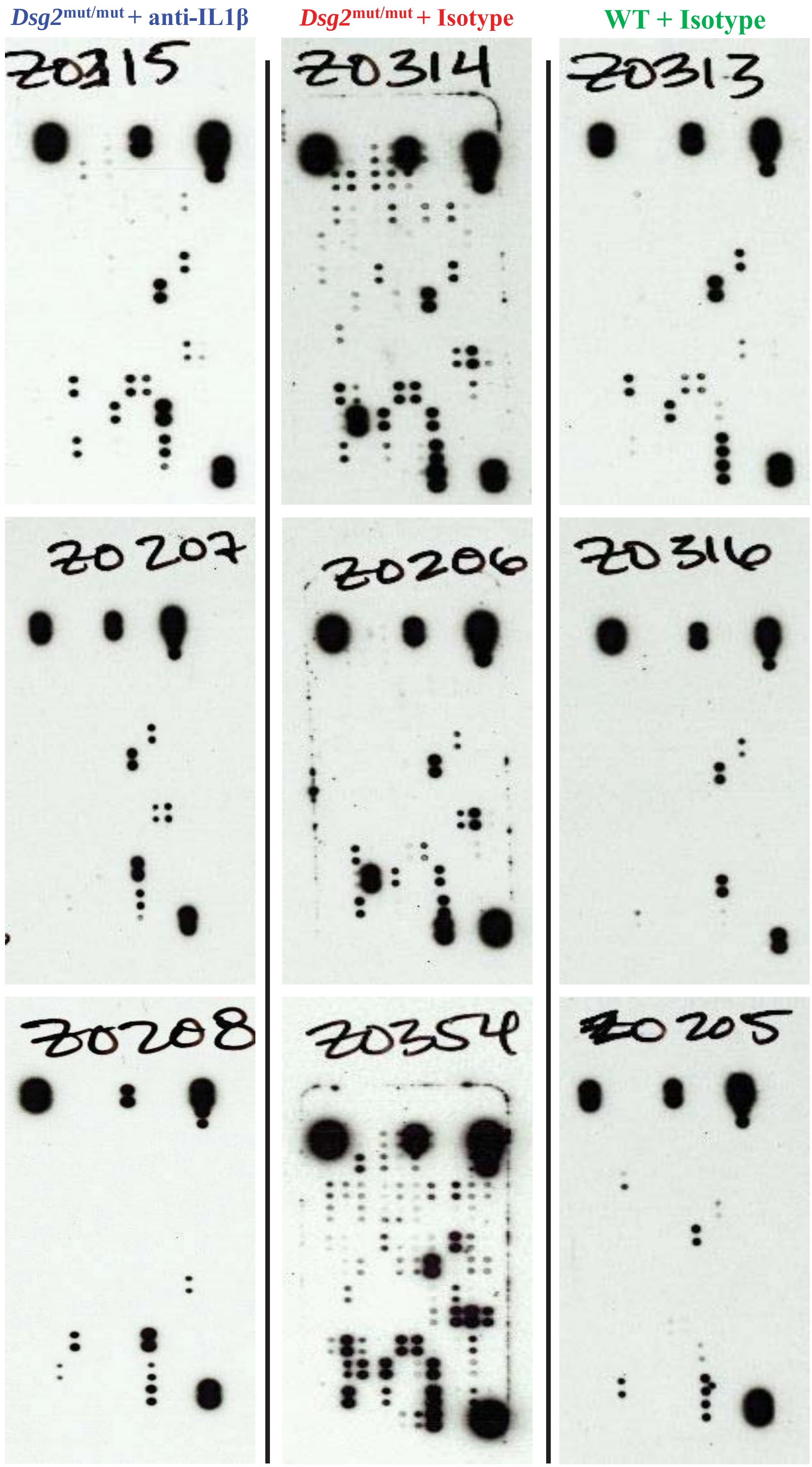

Dsg2 mut/mut + anti-IL1β
Dsg2 mut/mut + Isotype
WT + Isotype
20315
20314
20313
20207
20206
20316
20208
20354
20205

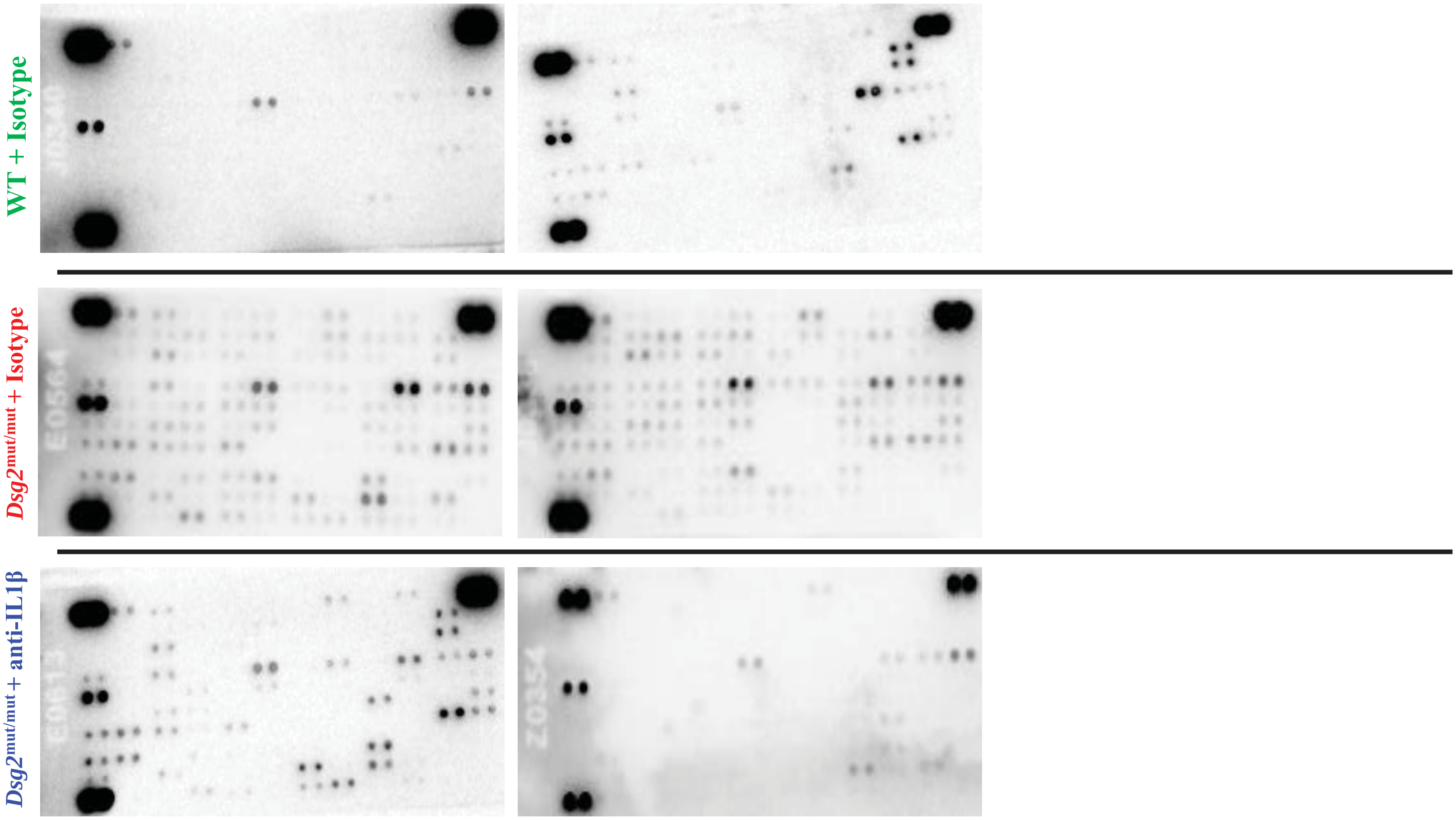
WT + Isotype
Dsg2mut/mut + Isotype
Dsg2mut/mut + anti-IL1β
